# Diet-Associated Regulation of Cardiac Metabolism: Molecular Determinants and Pathophysiological Consequences

**DOI:** 10.3390/nu18152451

**Published:** 2026-07-27

**Authors:** Gaetano Pacinella, Anna Maria Ciaccio, Carlo Domenico Maida, Vittoriano Della Corte, Giuseppe Miceli, Mario Daidone, Cosimo Quaranta, John Sebastian Soldano, Antonino Tuttolomondo

**Affiliations:** 1 Department of Promoting Health, Maternal-Infant, Excellence, and Internal and Specialised Medicine (PROMISE) “G. D’Alessandro”, University of Palermo, 90127 Palermo, Italy; pacinella66@gmail.com (G.P.); carlodomenico.maida@hotmail.com (C.D.M.); vittoriano.dc@gmail.com (V.D.C.); miceli.gpp@gmail.com (G.M.); mario.daidone@unipa.it (M.D.);; 2 Internal Medicine and Stroke Care Ward, Policlinico “P. Giaccone”, 90127 Palermo, Italy

**Keywords:** cardiac metabolism, metabolic flexibility, diet, myocardial energetics, nutrient sensing, mitochondrial dysfunction, lipotoxicity, glucotoxicity, gut–heart axis, cardiac remodelling, heart failure, precision nutrition

## Abstract

The heart is a highly energy-demanding organ that depends on metabolic flexibility to adjust substrate utilization in response to changes in nutrient availability, endocrine signals, and energetic demands. Accumulating evidence demonstrates that dietary patterns are key determinants of myocardial metabolic homeostasis, affecting substrate selection, mitochondrial function, nutrient-sensing pathways, and long-term transcriptional and epigenetic regulation. This review analyzes the molecular mechanisms through which diet regulates cardiac metabolism and explores how chronic nutritional exposures influence the myocardial energetic phenotype. The physiological regulation of cardiac substrate utilization is described, with emphasis on fatty acids, glucose, ketone bodies, and branched-chain amino acids, underscoring the importance of metabolic flexibility in sustaining cardiac efficiency. The regulation of substrate transport and oxidation is examined, including the roles of the carnitine shuttle, insulin signaling, AMPK, mTOR, PPARα–PGC-1α, SIRT3, and other nutrient-sensing networks that coordinate mitochondrial ATP production. The effects of dietary composition and meal timing, such as caloric restriction and intermittent fasting, are discussed as modulators of myocardial metabolism. The adverse effects of chronic nutrient excess are reviewed, including lipotoxicity, glucotoxicity, insulin resistance, mitochondrial dysfunction, oxidative stress, pseudo-hypoxia, fetal metabolic reprogramming, and maladaptive cardiac remodeling. Recent findings on the gut–heart axis, microbiota-derived metabolites, circadian regulation, and metabolic–epigenetic interactions are also considered. Overall, current evidence supports the view that diet is an important and potentially modifiable regulator of the cardiac metabolic phenotype. Advancing the understanding of diet–metabolism interactions may enable the development of targeted nutritional strategies to maintain metabolic flexibility, enhance cardiac bioenergetics, and prevent the progression of heart failure and other cardiometabolic diseases.

## 1. Cardiac Metabolism as a Diet-Sensitive System

The adult human heart contracts approximately 100,000 times per day and exhibits the highest energy consumption per gram among all organs. To maintain continuous contractile function, the myocardium has adapted as a metabolic omnivore, capable of oxidising fatty acids, glucose, lactate, ketone bodies, and amino acids in varying proportions depending on substrate availability, hormonal milieu, and workload [1].

Under resting and fasted conditions, 60–90% of cardiac acetyl-CoA originates from fatty acid β-oxidation, while the remainder is supplied by glucose, lactate, ketone bodies, and branched-chain amino acids (BCAAs). This substrate promiscuity, known as metabolic flexibility, represents a fundamental determinant of cardiac performance and resilience rather than a mere biochemical curiosity [2].

Circulating substrate concentrations are determined by dietary intake, positioning the heart at the downstream end of a metabolic pathway that originates with food consumption. Each meal modifies the arterial composition of fuels delivered to cardiomyocytes, while sustained dietary habits can induce lasting changes in the transcriptional, enzymatic, and transport systems that regulate myocardial substrate selection.

The expression “metabolic omnivore” originated from early coronary sinus catheterisation and isolated heart perfusion experiments in the mid-twentieth century, and encapsulates a fundamental biological principle: the heart is capable of degrading most energy-providing substrates in the presence of oxygen to produce ATP. When oxygen is unavailable, the heart continues to generate ATP through substrate-level phosphorylation, converting glucose to lactate and certain amino acids to succinate. This metabolic versatility is regulated by a multilayered hierarchy of allosteric, transcriptional, and post-translational mechanisms, allowing the heart to switch between fuel sources over timescales ranging from seconds to days [3].

### 1.1. Metabolic Flexibility as a Defining Feature of the Myocardium

Metabolic flexibility in the myocardium denotes the heart’s ability to rapidly switch between energy-producing substrates in response to fluctuations in substrate availability, hormonal milieu, oxygen supply, cardiac workload, and circadian rhythms. This adaptability is not a passive result of substrate diffusion but is governed by an actively regulated, multilayered biological program operating across multiple timescales, from seconds (allosteric enzyme modulation) to hours (post-translational modifications) to days and weeks (transcriptional reprogramming). This concept is fundamental to cardiac physiology, as the heart, unlike skeletal muscle or the brain, cannot withstand even brief interruptions in ATP supply. Myocardial ATP stores are extremely limited (~300 mg in a 300 g heart) compared to the rate of consumption (~30 mg/s), resulting in complete turnover of the ATP pool approximately every 10 s. Metabolic flexibility thus enables the heart to match ATP production with ATP demand on a beat-to-beat basis, irrespective of dietary intake, physical activity, or sleep–wake state [4].

Under fasting conditions, fatty acid β-oxidation provides 40–60% of cardiac acetyl-CoA, glucose supplies 20–40%, and ketone bodies and amino acids account for the remainder (10–15% and 1–2%, respectively). These proportions are dynamic: following a carbohydrate-rich meal, glucose can contribute approximately 70% of total substrate utilisation, whereas after a fat-rich meal, its contribution may decrease to as little as 10%. This nearly seven-fold variation in glucose utilisation, determined solely by meal composition, highlights the rapid influence of dietary intake on myocardial metabolism. Exercise increases skeletal muscle lactate production, resulting in a substantial rise in cardiac lactate oxidation and transforming a peripheral “waste product” into a major energy source. Fasting or carbohydrate restriction stimulates hepatic ketogenesis, elevating circulating β-hydroxybutyrate, and the heart increases ketone oxidation in direct proportion to arterial concentration. This relationship remains linear and does not reach saturation under physiological conditions. Such substrate flexibility prevents reliance on a single fuel source and provides the heart with resilience to fluctuations in nutrient availability [1].

**Figure 1:**
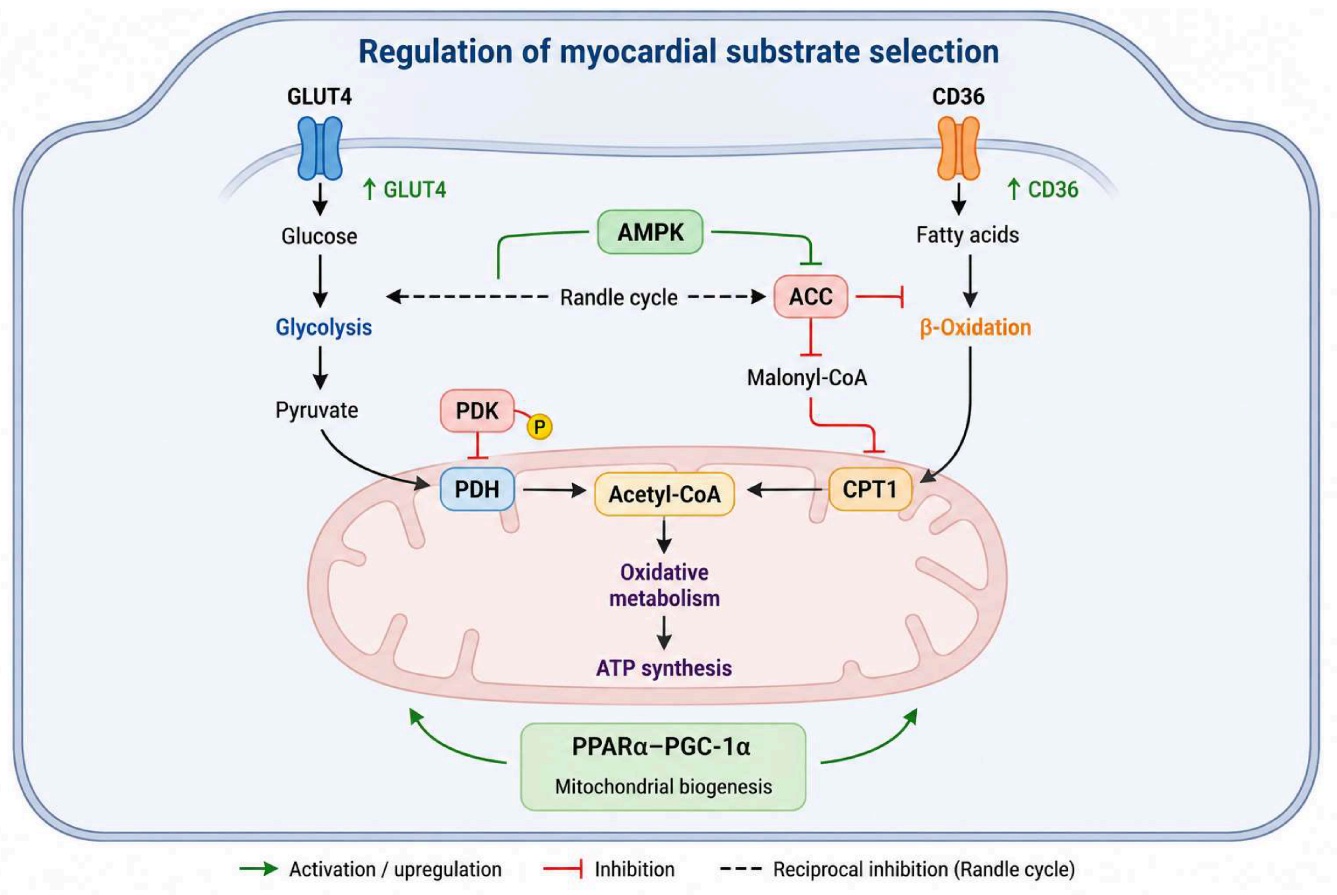
Schematic overview of substrate utilization in the cardiomyocyte: AMPK coordinates glucose and fatty acid metabolism through regulation of GLUT4, CD36, ACC, CPT1, PDH/PDK, SIRT3, and the PPARα–PGC-1α axis, integrating glycolysis, β-oxidation, mitochondrial ATP production, and metabolic gene expression. (AMPK: AMP-activated protein kinase; GLUT4: glucose transporter type 4; CD36: cluster of differentiation 36; ACC: acetyl-CoA carboxylase; CPT1: carnitine palmitoyltransferase 1; PDH: pyruvate dehydrogenase; PDK: pyruvate dehydrogenase kinase; SIRT3: sirtuin 3; PPARα: peroxisome proliferator-activated receptor alpha; PGC-1α: peroxisome proliferator-activated receptor gamma coactivator 1-alpha; ATP: adenosine triphosphate).

The main mechanism regulating substrate switching is the glucose–fatty acid cycle, originally described by Philip Randle in 1963 and subsequently improved through extensive cardiac-specific research (Figure 1). This mechanism operates through reciprocal inhibition mediated by shared mitochondrial intermediates. Increased fatty acid oxidation elevates mitochondrial acetyl-CoA/free CoA and NADH/NAD^+^ ratios, which activate pyruvate dehydrogenase kinase (PDK). PDK phosphorylates and inactivates the pyruvate dehydrogenase complex (PDH), thereby suppressing glucose oxidation. Concurrently, citrate exported from mitochondria inhibits phosphofructokinase-1 (PFK-1), slowing glycolysis and leading to glucose-6-phosphate accumulation. This accumulation inhibits hexokinase and reduces glucose uptake. Conversely, glucose availability suppresses fatty acid oxidation via the metabolic–epigenetic pathway. Acetyl-CoA carboxylase (ACC) converts acetyl-CoA to malonyl-CoA, which allosterically inhibits carnitine palmitoyltransferase I (CPT-I), the rate-limiting enzyme for mitochondrial fatty acid import. Malonyl-CoA concentration thus acts as a molecular rheostat, integrating glucose flux, energy status, and fatty acid availability into a unified regulatory signal that determines the balance between the two principal oxidative pathways [5].

AMP-activated protein kinase (AMPK) occupies a key position in this regulatory architecture. Activated by rising AMP/ATP ratios during energy stress (ischemia, exercise, hypoxia), AMPK phosphorylates and inhibits ACC, reducing malonyl-CoA synthesis and thereby relieving CPT-I inhibition—a process that promotes fatty acid oxidation when the heart is energy-depleted. Paradoxically, AMPK simultaneously stimulates glycolytic flux by activating phosphofructokinase-2 and increasing fructose-2,6-bisphosphate production, consequently accelerating both glycolysis and fatty acid oxidation while decreasing glucose oxidation (via PDH). This uncoupling of glycolysis from glucose oxidation during ischemia, with increased proton and lactate production, is a maladaptive consequence of AMPK activation that contributes to ischemic injury and has motivated medical strategies to recouple these pathways. Furthermore, AMPK and fatty acids synergistically induce PDK4 expression by modulating peroxisome proliferator-activated receptor alpha (PPARα) ligand-dependent activation, reinforcing transcriptional suppression of glucose oxidation during fasting and lipid excess [6].

An increasingly recognised aspect of metabolic flexibility regulation occurs at the level of substrate transporter trafficking. CD36 (fatty acid translocase) and GLUT4 (insulin-responsive glucose transporter) are the primary membrane transporters responsible for the entry of long-chain fatty acids and glucose into cardiomyocytes, respectively. Both transporters continuously recycle between intracellular endosomal compartments and the sarcolemma via vesicle-mediated processes, with their surface abundance dynamically regulated by insulin signalling, contraction-induced AMPK activation, and calcium-dependent mechanisms. Under physiological conditions, both insulin and contraction promote the translocation of CD36 and GLUT4 to the cell surface, thereby increasing the uptake of both substrates simultaneously [7].

Chronic lipid overload, such as that resulting from a Western diet, leads to a significant divergence in the trafficking of CD36 and GLUT4. CD36 is persistently relocated to the sarcolemma, while GLUT4 remains sequestered within endosomal compartments. This differential redistribution represents an early event in the development of myocardial insulin resistance and may contribute to subsequent metabolic disturbances, including lipotoxicity, impaired glucose oxidation, and reduced cardiac efficiency, all of which are characteristic of the diabetic heart. Recent studies have identified trafficking proteins, including vacuolar-type H^+^-ATPase and specific vesicle-associated membrane proteins (VAMPs), that are dedicated to either CD36 or GLUT4 trafficking [8]. These proteins represent potential therapeutic targets for selectively correcting abnormal substrate uptake while maintaining the function of the other transporter. Furthermore, protein kinase D1 (PKD1) and its downstream effector phosphatidylinositol 4-kinase IIIβ (PI4KIIIβ) have been identified as a signalling hub that specifically enhances GLUT4-mediated glucose uptake without inducing hypertrophy. Targeting this pathway may provide a promising strategy for restoring the fatty acid-to-glucose substrate balance in the metabolically inflexible heart [9].

Superimposed on the acute allosteric and trafficking-based controls is a slower, yet more durable, layer of transcriptional regulation that determines the heart’s long-term metabolic capacity. The PPARα–PGC-1α axis serves as the master transcriptional program for fatty acid oxidation, controlling the expression of CD36, CPT-I, the β-oxidation enzymes, and components of the electron transport chain. PPARα is activated by fatty acid ligands, and experimental evidence suggests that increased fatty acid delivery may promote a feed-forward transcriptional program that enhances the expression of fatty acid oxidation machinery, thereby increasing the heart’s capacity for lipid utilization.

PGC-1α, the obligate coactivator, is itself regulated by multiple signals, including cold exposure, exercise, fasting, and thyroid hormone—all conditions that increase fatty acid availability and cardiac energy demand. In heart failure and cardiac hypertrophy, the PPARα–PGC-1α axis is progressively downregulated, shifting the heart toward a “fetal” metabolic phenotype characterised by increased glycolysis and decreased fatty acid oxidation—a shift that is initially adaptive (improving oxygen efficiency) but ultimately maladaptive (creating an energy deficit that glycolysis alone cannot compensate) [10].

Post-translational modifications provide an additional layer of regulatory control. Acetylation, malonylation, succinylation, and glutarylation of mitochondrial metabolic enzymes modulate enzymatic activity in response to fluctuations in the cellular metabolic environment. The mitochondrial deacetylase SIRT3 is essential in this context.

In pathological states such as obesity and heart failure, diminished SIRT3 activity has been associated with hyperacetylation of key β-oxidation enzymes (LCAD, β-HAD) and malonyl-CoA decarboxylase. This hyperacetylation paradoxically enhances fatty acid oxidation and promotes metabolic rigidity. These post-translational mechanisms operate on intermediate timescales, from minutes to hours, and bridge the gap between rapid allosteric regulation and slower transcriptional responses [4].

Together, the Randle cycle, AMPK signaling, substrate-transporter trafficking, and the PPARα–PGC-1α transcriptional axis constitute the core regulatory framework of myocardial metabolic flexibility. These mechanisms are introduced here and are subsequently discussed only in relation to specific dietary or pathological contexts.

### 1.2. Diet as a Long-Term Regulator of Cardiac Metabolic State

A chronic dietary environment, defined by persistent patterns of macro- and micronutrient intake, modifies heart metabolism at transcriptional, epigenetic, and structural levels. Evidence from preclinical models, human metabolic monitoring, and large-scale epidemiological studies indicates that diet acts as a long-term regulator of cardiac metabolic state, using mechanisms beyond the acute substrate-switching dynamics described by the Randle cycle [11].

**Figure 2:**
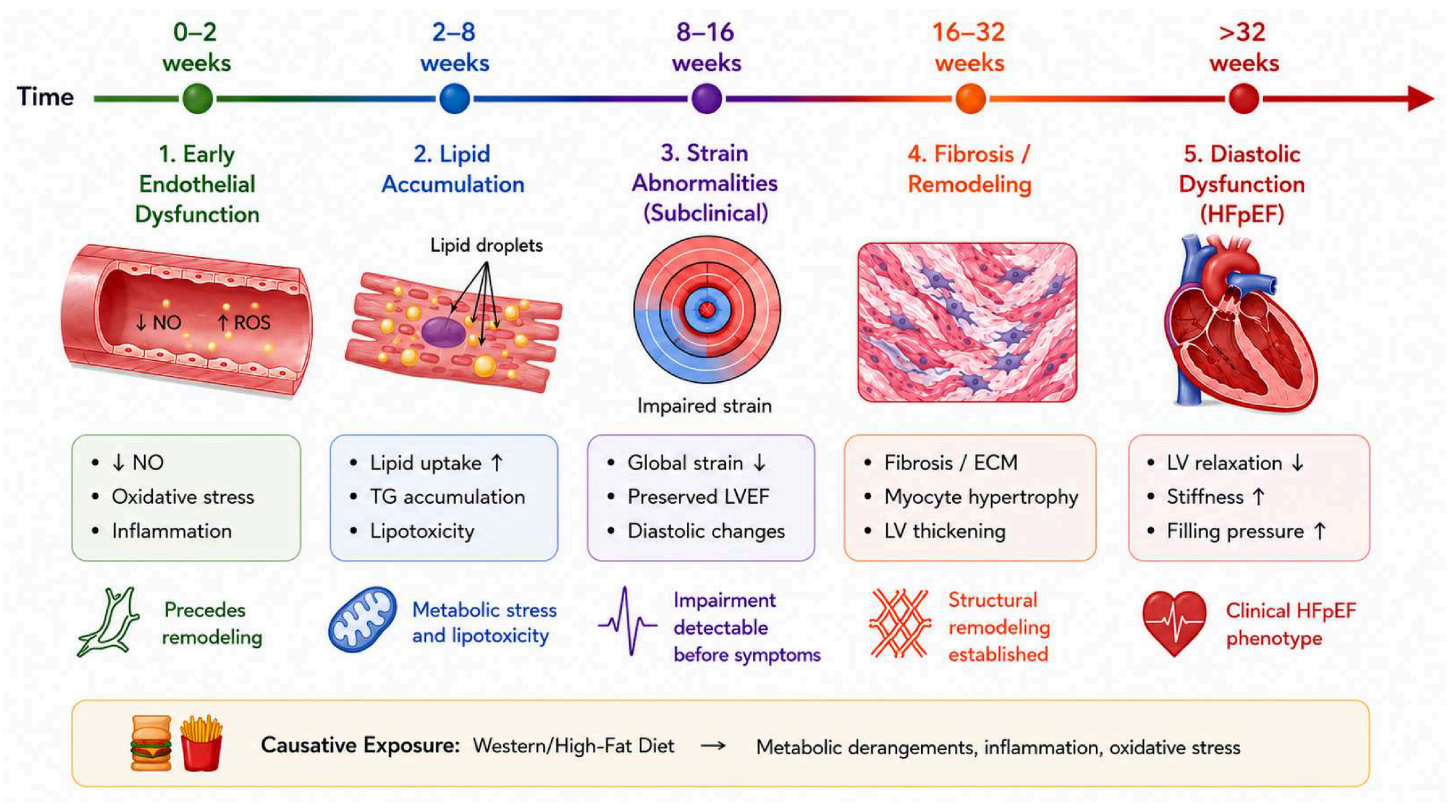
Temporal progression of Western/high-fat diet-induced cardiac remodeling. Early endothelial dysfunction and myocardial lipid accumulation precede subclinical impairment in myocardial strain, followed by fibrotic remodeling and increased ventricular stiffness. These alterations ultimately culminate in diastolic dysfunction and a heart failure with preserved ejection fraction (HFpEF) phenotype (LVEF: left ventricular ejection fraction; HFpEF: heart failure with preserved ejection fraction; ↑: increased; ↓: reduced).

The 2026 AHA Scientific Statement on Dietary Guidance to Improve Cardiovascular Health emphasizes the importance of heart-healthy dietary patterns over the consideration of individual foods or nutrients. It asserts that overall dietary patterns, encompassing all foods and beverages consumed daily, are the most comprehensive determinants of cardiovascular health (Figure 2). Similarly, the 2025 ACC Concise Clinical Guidance on Nutrition affirms the central role of nutrition in cardiovascular health. This guidance identifies saturated fats, added sugars, sodium, and ultra-processed foods as primary contributors to cardiovascular harm, while dietary fibre, plant-based proteins, potassium-rich foods, and omega-3 fatty acids are recognized as cardioprotective. Although these guidelines do not yet fully articulate this perspective, emerging basic science literature increasingly suggests that these dietary components influence cardiovascular health not only by modifying systemic risk factors, such as lipid levels, blood pressure, and glycemic control, but also by modulating substrate metabolism, mitochondrial function, and energetic efficiency [12].

The Western diet, characterised by high levels of saturated fat, refined sugar, and sodium, and low fibre intake, is the most extensively studied dietary pattern in relation to cardiac metabolic remodelling [13].

Rodents consuming a high-fat diet (45 to 60% of calories from fatty acids) for 8 to 10 weeks develop obesity, glucose intolerance, and systemic insulin resistance, accompanied by specific alterations in cardiac metabolism. These alterations include increased cardiac fatty acid oxidation, decreased glucose oxidation, and the development of cardiac insulin resistance. Importantly, these metabolic changes precede the onset of cardiac impairment, which typically appears 15 to 20 weeks after initiating a high-fat diet. This time-related sequence raises the question of whether metabolic reprogramming directly contributes to subsequent contractile dysfunction [3].

Recent studies have clarified the temporal sequence of these changes. Kwiatkowski et al. demonstrated, using cardiac MRI, that coronary microvascular endothelial dysfunction, indicated by reduced nitric oxide synthase-dependent vasodilation, was detectable as early as seven days after initiating a high-fat diet in mice. This dysfunction occurred prior to systemic insulin resistance and before any measurable change in systolic function. Increased lipid accumulation in cardiomyocytes was also observed within seven days and persisted at eight weeks [14].

Ternacle et al. further confirmed that early left ventricular dysfunction, assessed by radial strain rate imaging, was evident at five weeks of high-fat feeding and was associated with increased cardiomyocyte apoptosis and interstitial fibrosis. These pathological changes progressively worsened over a 20-week period [15].

A landmark 2026 study by Oka et al. in the Journal of Clinical Investigation identified a novel molecular mechanism linking dietary fat to diastolic dysfunction. Elevation of free fatty acids induced by high-fat diet consumption was shown to stimulate Interleukin-6 (IL-6) production in cardiomyocytes by forming a PPARα-NF-κB (Nuclear factor kappa-light-chain-enhancer of activated B cells) heterodimer that bound to the NF-κB element in the IL-6 promoter. This autocrine IL-6 production occurred before the development of local macrophage-mediated inflammation and was sufficient to induce features of diastolic dysfunction in this experimental model, even when macrophages were depleted [16].

Cardiac-specific deletion of either PPARα or IL-6 reduced high-fat diet-induced diastolic dysfunction, supporting the PPARα–NF-κB–IL-6 axis as a potential mechanistic link between dietary fat excess and cardiac functional impairment.

Liu et al. (2023) demonstrated that long-term Western diet feeding for seven months resulted in a cascade of cardiac microvascular dysfunction, disruption of mitochondria-associated endoplasmic reticulum membranes (MAM), mitochondrial shape transition and damage, and activation of caspase-dependent apoptosis pathways, finally leading to cardiac dysfunction and remodelling [17].

The type of dietary fat is also significant. Yamamoto et al. showed that saturated fatty acid-rich high-fat diets induced more severe diastolic dysfunction than monounsaturated fatty acid-rich diets, despite similar activation of myocardial fatty acid uptake, triglyceride turnover, and mitochondrial fatty acid oxidation. This difference was attributed to lowered membrane phospholipid unsaturation, induction of the unfolded protein response, and suppression of stearoyl-CoA desaturase-1 (SCD1) [18].

The sugar component of the Western diet exerts cardiac effects that are distinct from and additive to those of dietary fat. Ashraf et al. (2020) demonstrated that dietary fat and sugar differentially affect cardiac growth signalling pathways: high-fat diets primarily enhanced AKT phosphorylation through insulin resistance, while high-sugar diets potentiated β-adrenergic stimulation of the glucose-sensitive kinases Proline-rich tyrosine kinase 2 (PYK2) and Extracellular signal-regulated kinase (ERK), leading to increased phosphorylation of the protein synthesis regulator Ribosomal protein S6 kinase beta-1 (S6K1), with the Western diet (combining both) producing additive effects [19].

Fructose, the principal monosaccharide in added sugars and sugar-sweetened beverages, has emerged as a potential contributor to adverse cardiac metabolic remodeling. Unlike glucose, fructose bypasses the rate-limiting glycolytic enzyme phosphofructokinase and proceeds through glycolysis in an unregulated manner. Plasma and cardiac fructose levels are elevated in patients with diabetes, and cardiomyocytes express all necessary proteins for fructose transport and metabolism [20].

When dietary fructose intake is elevated and myocardial glucose uptake is compromised by insulin resistance, increased cardiomyocyte fructose flux may represent a metabolic challenge involving unregulated glycolysis, oxidative stress, lipid accumulation, and accelerated protein modifications, including O-GlcNAcylation and advanced glycation end product formation. Hepatic fructose metabolism further drives de novo lipogenesis, atherogenic dyslipidemia, and visceral adiposity—systemic effects that compound the direct myocardial toxicity [21].

**Figure 3:**
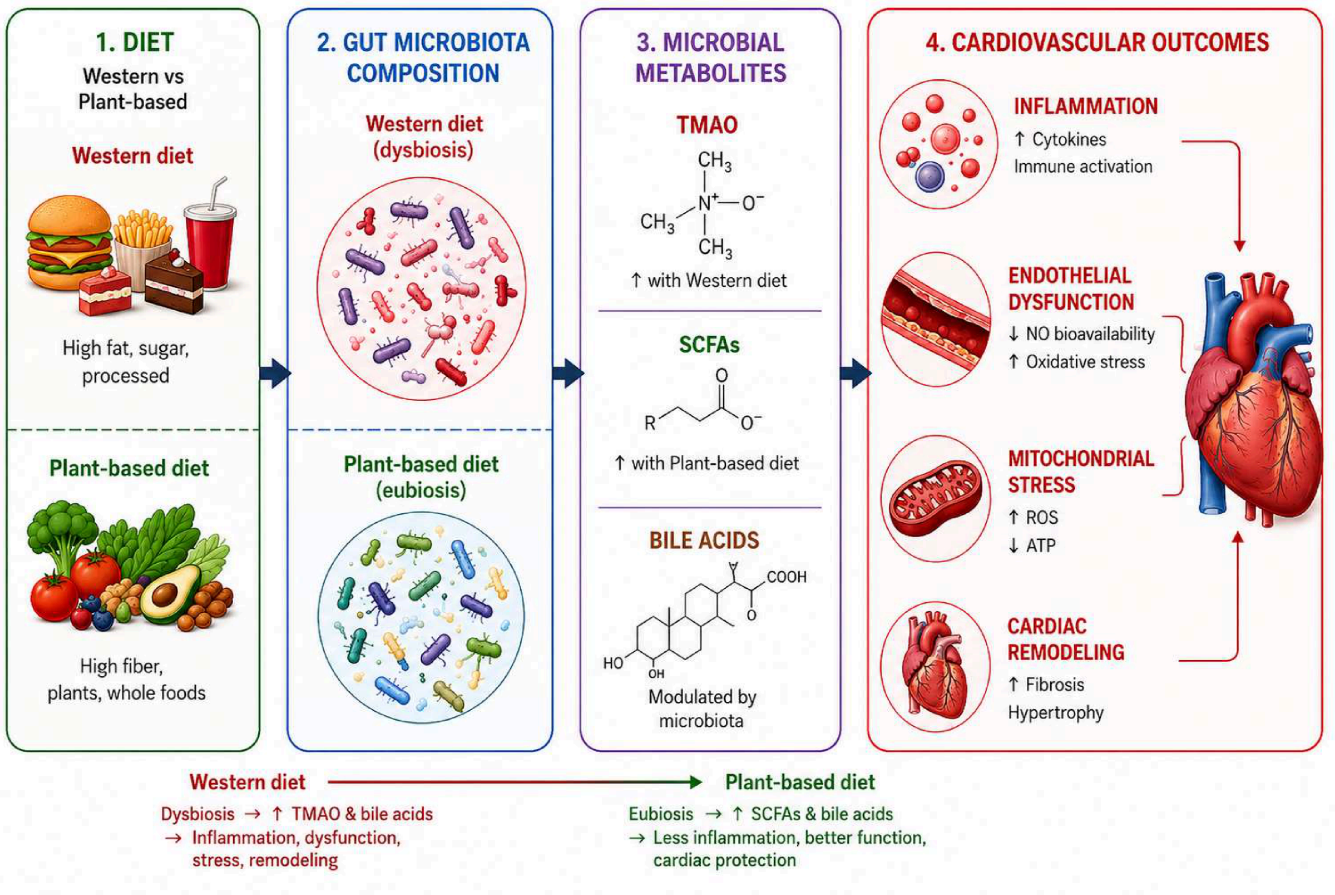
Gut–heart axis linking dietary patterns, gut microbiota, and cardiovascular remodeling. Western dietary patterns promote gut dysbiosis and increased production of trimethylamine N-oxide (TMAO) and pro-inflammatory bile acid profiles, whereas plant-based diets favor eubiosis and short-chain fatty acid (SCFA) production. These microbiota-derived metabolites influence inflammation, endothelial function, mitochondrial homeostasis, and ultimately cardiac remodeling and dysfunction. (TMAO: trimethylamine N-oxide; SCFAs: short-chain fatty acids; IL-6: interleukin-6; TNF-α: tumor necrosis factor-α; CRP: C-reactive protein; ROS: reactive oxygen species; ATP: adenosine triphosphate; ↑: increased; ↓: reduced).

A rapidly expanding dimension of diet-cardiac metabolism interaction operates through the gut microbiome (Figure 3). Experimental and clinical studies suggest that Western dietary patterns are associated with alterations in gut microbiota composition, promoting the generation of pathogenic metabolites—most notably trimethylamine-N-oxide (TMAO)—while simultaneously suppressing the production of beneficial metabolites, including short-chain fatty acids (SCFAs). The metabolic shift triggers systemic inflammatory responses, oxidative stress, and metabolic disturbances, accelerating cardiovascular disease progression [22].

TMAO, produced by hepatic oxidation of trimethylamine (TMA) generated by gut bacterial metabolism of dietary choline, L-carnitine, and phosphatidylcholine—nutrients abundant in red meat, eggs, and full-fat dairy—promotes macrophage activation, damages vascular endothelium, disrupts cholesterol metabolism, and is consistently associated with increased cardiovascular risk during observational studies [23].

Despite these associations, the role of TMAO in cardiovascular disease remains a subject of continuing debate. While numerous observational studies have identified elevated circulating TMAO concentrations as predictors of adverse cardiovascular outcomes, causality has not been definitively established in humans. Conflicting findings may reflect differences in eating habits, renal function, gut microbial composition, and analytical methodologies. Accordingly, TMAO should currently be regarded as an encouraging biomarker and potential mediator of cardiometabolic risk, although further mechanistic and interventional studies are required to clarify its direct pathogenic role. Conversely, SCFAs—particularly butyrate and propionate, produced by bacterial fermentation of dietary fibre—exert anti-atherosclerotic effects by promoting regulatory T cell differentiation, inhibiting histone deacetylases, and improving endothelial function. A 2026 comprehensive review by He et al. in Frontiers in Microbiology and a 2025 review by Kondapalli et al. in Comprehensive Physiology established that the gut–heart axis represents a bidirectional communication system in which dietary patterns reshape gut microbial communities, which in turn produce metabolites that directly modulate cardiomyocyte function, cardiac fibroblast activity, and systemic inflammation [24]. Collectively, the available evidence consistently supports the concept that dietary composition deeply influences myocardial metabolic homeostasis through interconnected effects on substrate availability, mitochondrial function, inflammation, and gut microbiota-derived signaling. However, the strength of evidence varies across mechanisms. Most mechanistic insights originate from experimental animal models employing heterogeneous dietary protocols that only partially reproduce the complexity of human dietary behaviors. In contrast, human studies are predominantly observational and therefore cannot fully establish causality. Future investigations integrating mechanistic metabolic phenotyping with controlled nutritional interventions will be essential to clarify the relative contribution of individual nutrients versus overall dietary patterns. Importantly, despite the limited availability of mechanistic studies in humans, the overall direction of these experimental findings is consistent with clinical evidence demonstrating that healthy dietary patterns are associated with lower cardiovascular risk and reduced incidence of heart failure.

## 2. Dietary Modulation of Myocardial Substrate Utilization

In addition to macronutrient composition, the timing of nutrient intake significantly influences cardiac fuel selection. During caloric restriction, fatty acid oxidation becomes the primary energy source. Unlike chronic high-fat feeding, caloric restriction preserves full insulin sensitivity in the heart, allowing carbohydrate oxidation rates to remain elevated during feeding periods. This persistent metabolic flexibility is associated with reduced hypertrophic signalling, decreased adverse remodelling following myocardial infarction, and enhanced ischemia tolerance. These effects are linked to AMPK activation, reduced mitochondrial acetylation, and increased autophagy.

Intermittent fasting (IF) induces dynamic bioenergetic remodelling that depends on fasting duration. A multi-omics study in mice subjected to 12 h, 16 h, or every-other-day fasting regimens for six months demonstrated that shorter IF protocols led to coordinated changes in lipid and amino acid metabolism. In contrast, longer fasting regimens showed an inverse relationship between fatty acid oxidation and immune processes [25].

Functional echocardiographic assessments indicated improved cardiac performance under stress. In humans, time-restricted eating with 4–10 h feeding windows consistently reduces blood pressure by approximately 4/2 mmHg. Additionally, a three-week alternate-day fasting intervention increased myocardial flow reserve and reduced oxygen consumption, indicating direct improvements in cardiac metabolic efficiency [26].

Although direct assessment of myocardial metabolic remodeling in humans remains technically challenging, these clinical observations support the translational relevance of experimental evidence by demonstrating measurable improvements in cardiovascular physiology following dietary interventions.

These temporal dietary strategies utilise the heart’s intrinsic circadian metabolic programming by aligning fatty acid oxidation with fasting and glucose utilisation with feeding, thereby supporting physiological substrate oscillations.

The therapeutic potential of dietary substrate modulation is most clearly demonstrated by sodium–glucose cotransporter 2 (SGLT2) inhibitors. By inducing renal glucosuria, these agents decrease systemic carbohydrate availability and shift substrate utilisation toward fatty acids, ketone bodies, and glucagon, thereby mimicking a fasting-like metabolic state. Ertugliflozin also modulates cardiac metabolism through AMPK–mTOR signaling, thereby attenuating endoplasmic reticulum stress, apoptosis, and fibrosis [27].

These pharmacological effects reproduce, in a controlled and sustained manner, the metabolic benefits observed with caloric restriction and intermittent fasting, thereby providing strong evidence that dietary modulation of myocardial substrate selection represents a therapeutically actionable biological principle. Beyond SGLT2 inhibitors, other therapeutic strategies increasingly exploit modulation of cardiac metabolism. Regular aerobic exercise enhances myocardial metabolic flexibility by promoting mitochondrial biogenesis, increasing oxidative capacity, improving insulin sensitivity, and facilitating substrate switching, consequently reinforcing many of the adaptive metabolic responses induced by healthy dietary patterns. Likewise, GLP-1 receptor agonists and dual GIP/GLP-1 receptor agonists have demonstrated substantial cardiometabolic benefits through weight reduction, improved insulin sensitivity, attenuation of systemic inflammation, and indirect optimisation of myocardial substrate utilisation. Although the direct myocardial metabolic effects of these agents remain incompletely characterised, accumulating evidence suggests that combining dietary interventions, structured exercise, and targeted metabolic therapies may represent an effective strategy to preserve myocardial energetic homeostasis and reduce the progression of cardiometabolic disease.

### 2.1. Regulation of Substrate Selection and Oxidation

Because the heart relies heavily on fatty acid oxidation for ATP production, the mechanisms regulating fatty acid utilisation are of critical physiological importance. While circulating fatty acids are efficiently absorbed by cardiomyocytes, their subsequent oxidation depends on effective transport into the mitochondrial matrix, where β-oxidation occurs. Consequently, mitochondrial fatty acid trafficking constitutes a key regulatory checkpoint in cardiac energy metabolism.

**Figure 4:**
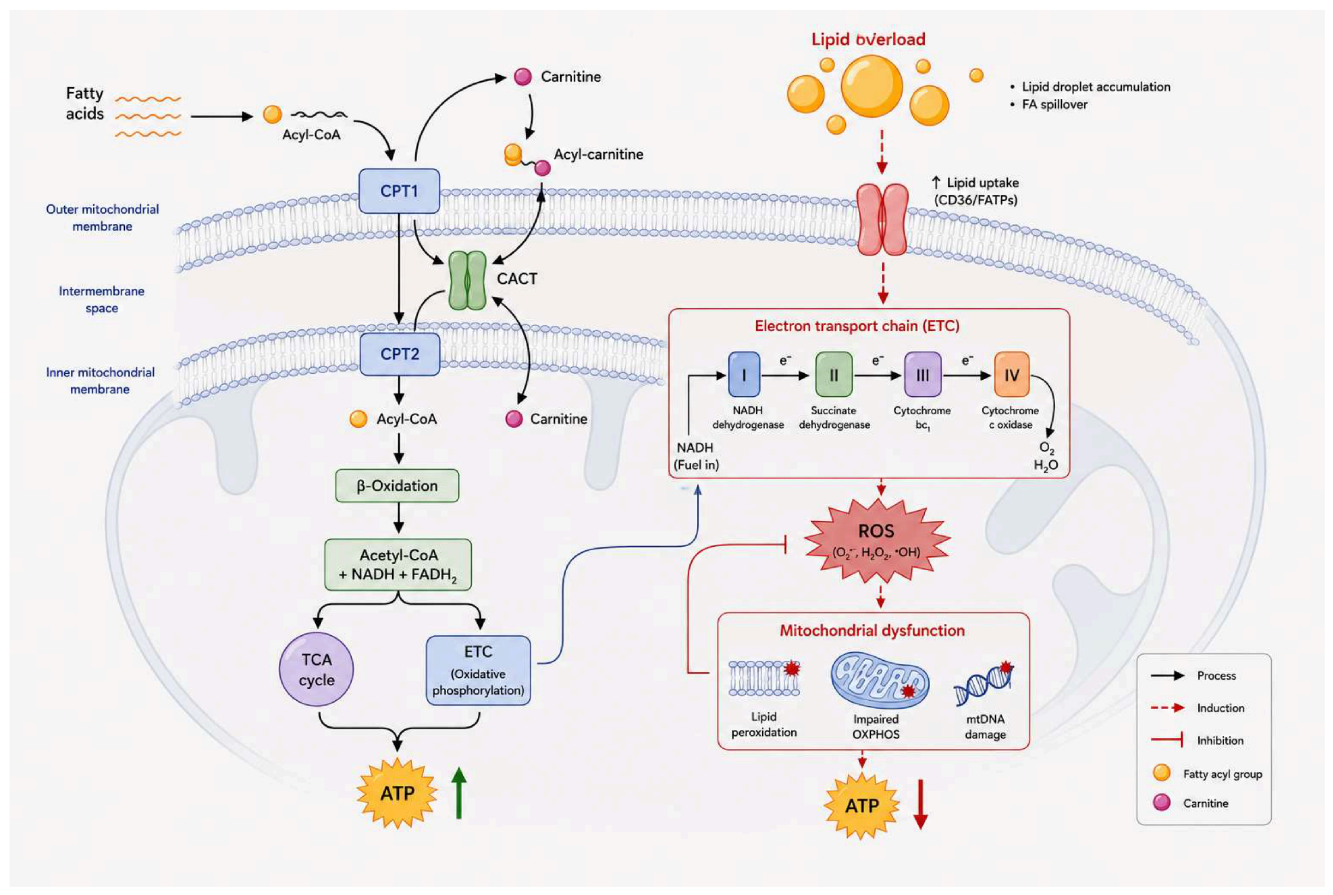
Overview of the carnitine shuttle and the molecular consequences of lipid overload in cardiomyocytes. Under physiological conditions, long-chain fatty acids are transported into mitochondria through the carnitine shuttle (CPT1–CACT–CPT2) to undergo β-oxidation, generating acetyl-CoA, NADH, and FADH_2_ that fuel the tricarboxylic acid (TCA) cycle and oxidative phosphorylation, thereby supporting ATP production. In conditions of lipid overload, increased fatty acid uptake and spillover enhance electron flux through the electron transport chain (ETC), promoting reactive oxygen species (ROS) generation. Excessive ROS induce mitochondrial dysfunction, impair oxidative phosphorylation, and ultimately reduce ATP production (ATP, adenosine triphosphate; CACT, carnitine-acylcarnitine translocase; CPT1, carnitine palmitoyltransferase 1; CPT2, carnitine palmitoyltransferase 2; ETC, electron transport chain; FA, fatty acid; FADH_2_, reduced flavin adenine dinucleotide; NADH, reduced nicotinamide adenine dinucleotide; OXPHOS, oxidative phosphorylation; ROS, reactive oxygen species; TCA, tricarboxylic acid; ↑, increase; ↓, reduction).

Long-chain fatty acyl-CoA esters are unable to freely cross the inner mitochondrial membrane. Instead, they are transported via a three-step shuttle system. First, CPT-1 (with CPT1B as the cardiac isoform) catalyses the transfer of the acyl group from CoA to carnitine on the outer mitochondrial membrane. Next, carnitine–acylcarnitine translocase (CACT) transports the resulting acylcarnitine across the inner membrane. Finally, CPT-2 reconverts acylcarnitine to acyl-CoA within the mitochondrial matrix, releasing free carnitine for recycling (Figure 4) [28].

This shuttle represents the principal rate-limiting checkpoint for long-chain fatty acid oxidation. Its regulation, primarily through malonyl-CoA-mediated inhibition of CPT1B, determines the rate at which fatty acids enter the β-oxidation pathway.

The physiological significance of CPT1B malonyl-CoA sensitivity was demonstrated by van Weeghel et al. (2018) using a knock-in mouse model expressing a CPT1B mutant with reduced malonyl-CoA sensitivity [29]. The 2025 Pharmacological Reviews article by Rodríguez-Rodríguez et al., the first comprehensive review of the CPT enzyme family from the International Union of Basic and Clinical Pharmacology, further established that CPT1B functions as an allosteric integrator rather than a passive gatekeeper. Its sensitivity to malonyl-CoA is modulated by membrane lipid composition, the ratio of palmitoyl-CoA to carnitine, and protein–protein interactions with the outer mitochondrial membrane voltage-dependent anion channel (VDAC). This creates a multi-input regulatory node that integrates information about lipid availability, energy status, and mitochondrial membrane potential [30].

The carnitine palmitoyltransferase (CPT) system constitutes a critical point of bioenergetic vulnerability. During chronic lipid excess, elevated fatty acid flux through CPT-1 induces futile cycling, wherein fatty acids are repeatedly transported into and out of mitochondria via the carnitine system, and intracellular triglycerides are subject to ongoing breakdown and resynthesis. These processes consume oxygen without generating contractile work. In conjunction with the activation of uncoupling protein-3 (UCP-3) by fatty acid metabolites, such futile cycles explain the observation that fatty acid oxidation increases cardiac oxygen consumption by 30–50% compared to glucose oxidation at equivalent stroke work indices. This non-contractile oxygen consumption substantially contributes to the reduced myocardial efficiency observed in diabetic and obese hearts, thereby elucidating why the oxygen cost of metabolic inflexibility exceeds predictions based solely on the phosphorylation-to-oxygen (P/O) ratio [1].

Concerning carbohydrate utilization, Insulin is the principal hormonal signal that promotes myocardial glucose uptake and utilisation. Binding of insulin to its receptor activates the PI3K–Akt signalling cascade, which stimulates translocation of GLUT4 from intracellular vesicles to the sarcolemma, thereby increasing glucose entry into the cardiomyocyte. Simultaneously, insulin promotes glycolysis and glycogen synthesis while modulating fatty acid uptake through parallel effects on CD36 trafficking. The Phosphoinositide 3-kinase (PI3K)–Akt pathway also exerts trophic effects—regulating protein synthesis, cell survival, and vascular tone—that extend insulin’s role beyond simple substrate regulation into broader cardiac homeostasis.

A critical feature of cardiac insulin signalling is its rapid adaptability to the systemic metabolic milieu. Under conditions of insulin resistance, the same processes that impair insulin action in skeletal muscle also occur in the myocardium: intracellular diacylglycerol accumulation, activation of serine kinases that phosphorylate and impair insulin receptor substrate-1 (IRS-1), and NFκB-mediated inflammation collectively reduce insulin’s ability to stimulate glucose uptake and oxidation. The resulting loss of insulin-mediated substrate flexibility—the inability to shift from fatty acid to glucose oxidation when metabolically appropriate—is a hallmark of the diabetic heart and a key contributor to the development of diastolic dysfunction and HFpEF. The sympathetic nervous system exerts rapid control over cardiac substrate selection through catecholamine-mediated activation of β-adrenergic receptors. Acute β_1_-adrenergic stimulation increases cardiac contractility and heart rate via Cyclic adenosine monophosphate (cAMP)/Protein kinase A (PKA) signalling, thereby elevating both energy demand and the mobilisation of metabolic substrates. Hepatic glycogenolysis releases glucose, while adipocyte lipolysis increases circulating free fatty acids. As a result, acute adrenergic activation simultaneously augments substrate supply and energy demand [31]. The specific substrate utilised depends on the relative concentrations of glucose and fatty acids, as well as the oxygen availability to the myocardium.

At the intracellular level, cAMP/PKA signalling regulates glycolytic flux by phosphorylating 6-phosphofructo-2-kinase (PFK-2), thereby increasing the production of fructose-2,6-bisphosphate (Fru-2,6-P_2_), a potent allosteric activator of PFK-1, the rate-limiting enzyme of glycolysis. Depre et al. demonstrated in isolated working rat hearts that increased workload led to a 50% reduction in the Km of PFK-2 for fructose-6-phosphate. This effect resulted from phosphorylation by either PKA or Ca^2+^/calmodulin-dependent kinase, establishing a mechanistic link between adrenergic stimulation, excitation–contraction coupling, and glycolytic activation. In contrast, chronic adrenergic activation, as observed in heart failure, shifts the metabolic response from adaptive to maladaptive [32]. Prolonged β-adrenergic stimulation downregulates PPARα/PGC-1α signalling, reduces mitochondrial biogenesis, and impairs fatty acid oxidation capacity. Concurrently, increased substrate delivery from peripheral lipolysis creates a mismatch between fatty acid supply and oxidative capacity, promoting intracellular lipid accumulation.

The bifunctional enzyme 6-phosphofructo-2-kinase/fructose-2,6-bisphosphatase (PFKFB), specifically the cardiac isoform PFKFB2, plays a central role in regulating cardiac glycolysis. Lawson and Uyeda (1987) demonstrated that, in perfused rat hearts, steady-state glycolytic rates correlated most closely with changes in Fru-6-P concentration and Fru-2,6-P_2_ levels, rather than with changes in ATP, citrate, AMP, or cytosolic phosphorylation potential [33]. These findings establish Fru-2,6-P_2_ as the primary physiological regulator of cardiac PFK-1 activity [33].

A 2025 study by Harold et al. in the Journal of the American Heart Association presented the first comprehensive characterization of cardiac-specific PFKFB2 knockout mice, demonstrating that PFKFB2 is essential for metabolic flexibility and differential glucose utilization. Loss of PFKFB2 resulted in moderate impairment of mitochondrial metabolic flexibility, which affected downstream glucose oxidation and respiration. Notably, PFKFB2 knockout hearts exhibited increased O-GlcNAcylation in the fed state, directly linking glycolytic flux regulation to the hexosamine biosynthetic pathway. Furthermore, PFKFB2 deficiency was sufficient to alter systemic circulating glucose levels under both fasted and stressed conditions, indicating that cardiac glycolytic regulation has systemic metabolic effects [34]. Wang et al. also demonstrated that reduced cardiac Fru-2,6-P_2_ levels intensified pressure overload-induced hypertrophy, fibrosis, and cardiac dysfunction, as evidenced by lower phosphocreatine-to-ATP ratios and increased oxidative stress. These findings suggest that the ability to elevate Fru-2,6-P_2_ during hemodynamic stress confers cardioprotection [35].

A small fraction (2–5%) of glucose entering the cardiomyocyte is diverted from glycolysis into the hexosamine biosynthetic pathway (HBP), whose rate-limiting enzyme is glutamine:fructose-6-phosphate amidotransferase 1 (GFAT1). The HBP produces UDP-N-acetylglucosamine (UDP-GlcNAc), the substrate for O-linked N-acetylglucosamine (O-GlcNAc) protein modification-a rapidly reversible post-translational modification that, like phosphorylation, modulates the activity of thousands of intracellular proteins but is regulated by only two enzymes: O-GlcNAc transferase (OGT) and O-GlcNAcase (OGA). 

The HBP functions as a critical sensor of nutrient surplus in the heart. Packer et al. in the European Journal of Heart Failure described how UDP-GlcNAc, acting in concert with mTOR and Hypoxia-inducible factor 1 alpha (HIF-1α), drives the recapitulation of fetal metabolic programming in the failing heart: prolonged increases in glucose uptake under stress increase HBP flux, heighten O-GlcNAcylation, and promote impaired calcium kinetics, contractile derangements, mitochondrial dysfunction, and maladaptive hypertrophy [36]. Laczy et al. (2011) demonstrated a direct link between HBP activation and substrate selection: glucosamine-induced increases in UDP-GlcNAc and O-GlcNAcylation in isolated rat hearts stimulated palmitate oxidation while decreasing lactate and pyruvate oxidation, an effect mediated at least in part by increased membrane levels of the fatty acid transporter CD36—suggesting that O-GlcNAcylation of CD36 may represent a novel glucose-dependent mechanism for redirecting cardiac metabolism toward fatty acid utilisation [37].

The double role of the HBP—acutely cardioprotective but chronically maladaptive—was comprehensively reviewed by Cairns et al. (2022): transient HBP activation during ischemia or acute stress enhances cell survival, whereas chronic upregulation (as in diabetes and heart failure) contributes to the onset and progression of cardiometabolic disease [38].

Branched-chain amino acids (BCAAs—leucine, isoleucine, and valine) have emerged as important modulators of cardiac substrate selection, extending beyond their traditional role as protein building blocks. The heart actively oxidises BCAAs, and impaired BCAA catabolism—whether genetic or acquired—has direct consequences for cardiac fuel selection.

Li et al. further elucidated the mechanism by which BCAAs upregulate fatty acid oxidation: valine and leucine, along with their corresponding branched-chain keto acid (BCKA) derivatives, transcriptionally upregulate PPARα through the General control nonderepressible 2 (GCN2)/Activating transcription factor 6 (ATF6) pathway, thereby directly linking amino acid sensing to the transcriptional programming of fatty acid metabolism [39]. BCAAs also activate mTOR signalling, which can alter tissue growth and insulin sensitivity, and in humans with established heart failure, impaired BCAA oxidation is associated with myocardial insulin resistance. Pharmacological inhibition of branched-chain ketoacid dehydrogenase kinase (BDK)—a negative regulator of BCAA catabolism-improved cardiac BCAA catabolic disorders and ameliorated post-infarction cardiac dysfunction and remodelling, supporting the therapeutic potential of taargeting BCAA metabolism in heart disease [40].

The discovery that the heart produces natriuretic peptides (Atrial natriuretic peptide-ANP-and B-type natriuretic peptide-BNP) established it as an endocrine organ with metabolic regulatory functions that reach far beyond hemodynamic control. Beyond their well-known natriuretic, diuretic, and vasodilatory actions mediated through the particulate guanylyl cyclase A (pGC-A)/Cyclic guanosine monophosphate (cGMP) pathway, natriuretic peptides activate lipolysis, promote lipid oxidation, stimulate mitochondrial respiration, and enhance white adipose tissue browning—collectively promoting a metabolic state that favours efficient oxidative metabolism [41].

The metabolic significance of natriuretic peptides is underscored by the observation that their deficiency is a consistent feature of obesity and type 2 diabetes, in contrast to the exaggerated release seen in heart failure [42]. Gupta and Wang (2015) further established that genetic and acquired deficiencies of the natriuretic peptide system can promote hypertension, cardiac hypertrophy, obesity, diabetes, and heart failure—positioning the natriuretic peptide axis as a bidirectional link between cardiac mechanical stress and systemic metabolic homeostasis [43].

A dimension of substrate regulation that has received increasing attention is the circadian clock—an autonomous molecular timekeeping mechanism within cardiomyocytes that temporally partitions metabolic functions across the 24 h day. The cardiomyocyte circadian clock, operating through the core clock components Brain and muscle ARNT-like protein 1 (BMAL1), Circadian locomotor output cycles kaput (CLOCK), nuclear receptor subfamily 1 group D members 1 and 2 (REV-ERBα/β), and period and cryptochrome proteins (PER/CRY), orchestrates daily rhythms in both oxidative and non-oxidative metabolism: cardiac glucose utilization peaks during the active period to meet increased energetic demands, while synthesis of glycogen and triglyceride peaks during the latter half of the active period in anticipation of the upcoming sleep/fasting phase, and protein turnover increases at the beginning of the sleep phase to promote growth and repair. These anticipatory metabolic oscillations confer a competitive advantage by allowing the heart to prepare for predictable changes in energy demand and nutrient supply before they occur—a fundamentally different regulatory strategy from the reactive mechanisms of the Randle cycle.

The circadian clock also interacts with the metabolic regulatory network at multiple levels. Metabolic oscillations in energy charge, redox status (NAD^+^/NADH ratio), reactive oxygen species, and O-GlcNAcylation feed back to modulate the clock mechanism itself, creating a two-way relationship in which the clock drives metabolic rhythms and metabolic state tunes the clock. This two-way regulation means that disruption of metabolic homeostasis—as occurs in diabetes, obesity, or heart failure—can desynchronize the circadian clock, and circadian disruption may in turn exacerbate metabolic dysfunction, forming a vicious cycle with immediate implications for cardiovascular disease in shift workers and in modern societies characterised by irregular eating and sleeping patterns [44].

### 2.2. Consequences of Nutrient Excess and Imbalance

**Figure 5:**
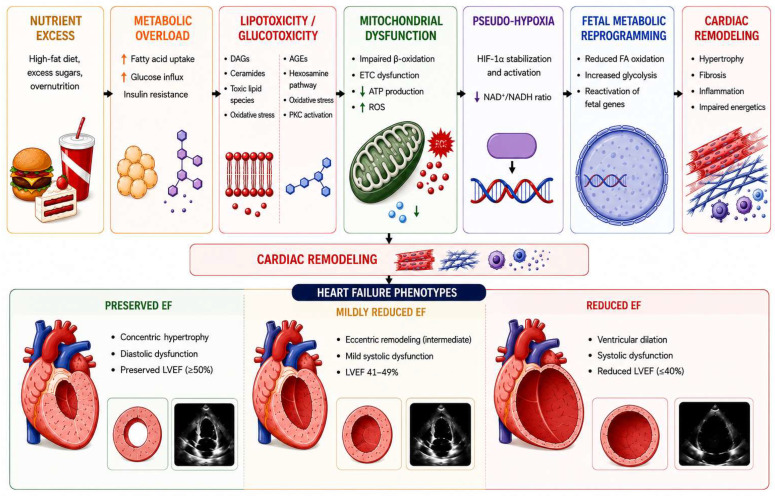
Excess nutrient availability promotes metabolic overload characterized by increased fatty acid and glucose flux, leading to lipotoxicity and glucotoxicity. These alterations converge on mitochondrial dysfunction, pseudo-hypoxia, and fetal metabolic reprogramming, ultimately driving adverse cardiac remodeling. Progressive structural, inflammatory, and energetic abnormalities contribute to the development of distinct heart failure phenotypes (DAGs: diacylglycerols; AGEs: advanced glycation end-products; ROS: reactive oxygen species; ETC: electron transport chain; ATP: adenosine triphosphate; HIF-1α: hypoxia-inducible factor-1 alpha; NAD+: oxidized nicotinamide adenine dinucleotide; NADH: reduced nicotinamide adenine dinucleotide; FA: fatty acid; ANP: atrial natriuretic peptide; BNP: B-type natriuretic peptide; β-MHC: beta-myosin heavy chain; HFpEF: heart failure with preserved ejection fraction; HFmrEF: heart failure with mildly reduced ejection fraction; HFrEF: heart failure with reduced ejection fraction; LVEF: left ventricular ejection fraction; ↑: increased; ↓: reduced).

When nutrient supply chronically exceeds energetic demand—as occurs in obesity, type 2 diabetes, and the metabolic syndrome—the regulatory systems described in the preceding sections become progressively dysregulated, initiating a cascade of maladaptive events that culminate in metabolic inflexibility, energetic inefficiency, and cardiac dysfunction (Figure 5). This section focuses on the pathological consequences arising specifically from this dysregulation: lipotoxic and glucotoxic injury, disruption of nutrient-sensing signalling networks, pseudo-hypoxia, reactivation and epigenetic entrenchment of fetal metabolic programming, and compensatory shift toward ketone oxidation.

Although the relative contribution of individual substrates differs across HFpEF, HFrEF, ischemic heart disease, and diabetic cardiomyopathy, impaired myocardial metabolic flexibility is a shared feature of these conditions. The following paragraphs therefore focus on the molecular consequences of chronic nutrient excess rather than reiterating disease-specific patterns of substrate utilization.

CD36 functions as a key gatekeeper of myocardial lipid metabolism, regulating cardiomyocyte fatty acid uptake and contributing to the development of metabolic disease when chronically overexpressed or dysregulated [45]. The principal mediators of this lipotoxic injury are ceramides, diacylglycerols (DAGs), and long-chain acylcarnitines, each exerting distinct but synergistic pathological effects.

Ceramides function as nutrient sensors of fatty acid excess. Mechanistically, ceramides inhibit Akt/PKB (Protein kinase B) through activation of protein phosphatase 2A (PP2A), perturb mitochondrial function by increasing Dynamin-related protein 1 (DRP1)-mediated fission, and promote apoptosis through both internal and external pathways. Choi et al. (2021) in Nature Reviews Cardiology demonstrated that pharmacological inhibition of de novo ceramide synthesis prevents the development of diabetes, atherosclerosis, and heart failure in preclinical models [46]. Law et al. (2018) further showed that very-long-chain ceramides derived from Ceramide synthase 2 (CerS2) are particularly lipotoxic, causing mitochondrial dysfunction and oxidative stress in a chain-length-dependent manner [47].

DAGs activate novel Protein kinase C (PKC) isoforms (PKCθ, PKCε) that phosphorylate IRS-1 at inhibitory serine residues, while palmitoylcarnitine and palmitoyl-CoA accumulation increase ventricular irritability and promote pathological left ventricular remodelling [48].

Chronic hyperglycemia exerts toxic effects through mechanisms distinct from those of the hexosamine biosynthetic pathway. Advanced glycation end-products (AGEs) are generated via non-enzymatic glycation of proteins, lipids, and nucleic acids. This process activates Receptor for advanced glycation end-products (RAGE)-mediated NFκB inflammatory signalling, increases collagen cross-linking and myocardial stiffness, and promotes microvascular dysfunction by inducing hyaline arteriolosclerosis in small coronary vessels. Each 1% increase in Glycated hemoglobin (HbA1c) correlates with an 8% higher risk of heart failure.

The polyol pathway, in which aldose reductase converts excess glucose to sorbitol and consumes Nicotinamide adenine dinucleotide phosphate, reduced form (NADPH), depletes cellular antioxidant reserves and heightens susceptibility to oxidative damage. Marwick et al. (2018) in JACC highlighted that AGE formation and persistent O-GlcNAc modification represent two distinct but converging detrimental pathways of glucose metabolism, both leading to epigenetic alterations and mitochondrial injury [49].

The molecular consequences of chronic nutrient excess converge on a fundamental imbalance between two opposing signalling networks: the nutrient-surplus pathway (PI3K–Akt–mTOR) and the nutrient-deprivation pathway (SIRT1–AMPK–PGC-1α). In the healthy heart, cardiomyocyte stress is minimised by the dominance of nutrient deprivation signalling: SIRT1, AMPK, and PGC-1α enhance oxidative metabolism and promote autophagy—the lysosome-dependent pathway that clears dysfunctional organelles. In the failing heart and in the heart subjected to chronic nutrient excess, this state inverts: enhanced PI3K–Akt–mTOR signalling promotes anabolism, prohypertrophic growth, and suppression of autophagy, while suppression of SIRT1/AMPK/PGC-1α impairs mitochondrial biogenesis and cytoprotective responses. Packer (2020) in the European Heart Journal established that reversal of this disproportion lessens the development of heart failure [50].

In obesity, HIF-1α is paradoxically stabilised under normoxic conditions—a state termed pseudo-hypoxia—driven by adipose tissue hypoxia, succinate-mediated prolyl hydroxylase domain protein (PHD) inhibition, and ROS-mediated stabilisation [51]. Warbrick and Rabkin (2019) proposed that HIF-1α may be a key factor in the development of HFpEF in obesity: increased HIF-1α expression in epicardial and myocardial adipose tissue drives a profibrotic transcriptional program (collagen I, III, IV, TIMP, lysyl oxidase) while recruiting M1 macrophages that mediate obesity-associated inflammation [52].

Conversely, in established type 2 diabetes, the capacity to activate HIF-1α in response to genuine hypoxia is impaired, creating vulnerability to ischemic injury. Sousa Fialho et al. (2021) demonstrated that pharmacological HIF-1α stabilisation with molidustat rescued cardiac metabolic dysfunction in diabetes, promoting glucose metabolism, suppressing fatty acid oxidation, and reversing impaired contractile recovery [53]. This dual derangement—inappropriate normoxic activation driving fibrosis, combined with impaired hypoxic activation compromising ischemic adaptation—represents a particularly insidious consequence of chronic nutrient excess.

Under sustained metabolic stress, the adult heart reactivates a fetal-like metabolic gene program characterised by increased glycolysis, decreased fatty acid oxidation, and reduced respiratory chain activity. Packer proposed a unifying framework in which this recapitulation is driven by the same nutrient-surplus signals, including mTOR, HIF-1α, and O-GlcNAcylation, that regulate fetal cardiac development. While glycolytic metabolism is adaptive in the hypoxic intrauterine environment, it becomes maladaptive in the normoxic adult heart [36].

The clinical implication is that, once epigenetically entrenched, the fetal metabolic program may resist reversal even after correction of the upstream metabolic insult. This phenomenon may explain why some patients with heart failure fail to recover metabolic function despite normalisation of hemodynamic parameters.

The 2025 Nature Reviews Cardiology review by Mericskay et al. distinguished between HFrEF and HFpEF: in HFrEF, fatty acid β-oxidation is often suppressed, and glycolysis and ketone oxidation only partially compensate; in HFpEF associated with metabolic diseases, elevated glucose and lipid levels overwhelm normal metabolic pathways, leading to accumulation of harmful byproducts that impair mitochondrial and cellular function [54].

Overall, these findings indicate that myocardial substrate selection is regulated by highly integrated metabolic networks rather than isolated pathways. Nevertheless, several proposed mechanisms—including alterations in transporter trafficking, nutrient sensing, and metabolic signaling—have been characterized predominantly in genetically modified animal models or in vitro systems. Their quantitative contribution to human cardiac physiology and disease progression remains incompletely defined. A major challenge for future research will be to integrate molecular, metabolic, and clinical data in order to distinguish adaptive metabolic remodeling from mechanisms directly driving cardiac dysfunction. Although direct assessment of myocardial metabolic pathways in humans remains technically challenging, clinical studies employing metabolic imaging, circulating biomarkers, and dietary intervention trials generally support the concept that preservation of metabolic flexibility is associated with improved cardiovascular health (Table 1).

## 3. Molecular Integration of Nutrient Signals in the Heart

Over the past decade, a significant reconceptualisation has occurred: metabolic intermediates produced during catabolism are not solely sources of fuel but also strongly regulate intracellular signalling, protein function, gene transcription, and epigenetic modifications, thereby influencing the heart’s stress response. Ritterhoff and Tian in Nature Reviews Cardiology described this paradigm shift, highlighting that metabolic remodelling in the failing heart goes beyond compromised energy supply. The altered metabolic network generates metabolites that directly regulate signalling cascades, protein function, and gene expression [55]. Flam and Arany (2023) in Nature Cardiovascular Research detailed how intermediates from glycolysis, the TCA cycle, nucleotides, amino acids, fatty acids, and ketones modulate cardiac signalling via mechanisms such as protein–metabolite interactions and epigenetic modifications [56]. The AHA Scientific Statement on Assessing Cardiac Metabolism identified this dual capacity as a key emerging biological concept: intermediary metabolites actively participate in cell signalling, for example, by influencing the acetylation profile of proteins that govern essential cellular processes. Consequently, alterations in substrate availability or metabolic flux have effects that go beyond bioenergetics, potentially reprogramming the transcriptional and epigenetic topography of cardiomyocytes. Three metabolic currencies—acetyl-CoA, NAD^+^, and the CoA/acyl-CoA family—serve as principal integrative hubs, encoding the cell’s energetic state and translating it into coordinated changes in enzyme activity, gene expression, and cell fate. Acetyl-CoA, the final product of major catabolic pathways, acts as a central metabolic second messenger. Its compartmentalised pools (mitochondrial, cytosolic, nuclear) simultaneously drive the TCA cycle, allosteric regulation, protein lysine acetylation, and histone acetylation, thereby functioning as a metabolic rheostat for gene expression [57].

NAD^+^ reflects metabolic demand rather than abundance, linking energy metabolism to sirtuin-mediated deacylation, DNA repair, and circadian rhythms through its interaction with the Nicotinamide phosphoribosyltransferase (NAMPT)-driven salvage pathway and the molecular clock [58,59]. The wider CoA/acyl-CoA family encodes information about lipid trafficking, as demonstrated by Goldenberg et al. (2019) in Circulation, who found that acyl-CoA depletion in failing hearts redirects fatty acids toward cardiotoxic ceramide species instead of oxidation [60]. Beyond cell-autonomous mechanisms, TCA cycle intermediates, particularly succinate acting through G protein-coupled receptor 91 (GPR91), function as extracellular signalling molecules that connect mitochondrial function to systemic metabolic communication. Additionally, gut-derived metabolites such as TMAO, SCFAs, and bile acids extend this link to the gut–heart axis, translating dietary patterns into remote cardiac metabolic signals [61,62]. The following two subsections will address the nutrient-sensing kinase cascades that transduce these integrated metabolic signals and the transcriptional machinery that converts them into gene expression programs.

### 3.1. Nutrient-Sensing Pathways and Metabolic Signaling

Cardiomyocytes integrate signals derived from glucose, fatty acids, amino acids, and ketone bodies through interconnected nutrient-sensing pathways. Building on the metabolic functions described in Section 1.1, AMPK also coordinates broader cellular responses by inhibiting mTORC1, activating Unc-51-like kinase 1 (ULK1)-dependent autophagy, and promoting PGC-1α-mediated mitochondrial biogenesis. These actions extend its role beyond acute substrate selection to the regulation of cellular growth, survival, and quality-control processes [63,64].

The reciprocal antagonism between AMPK and mTORC1 constitutes the most fundamental organisational principle of the cardiac nutrient-sensing network. González et al. in Cell Metabolism characterised AMPK and mTOR as the “Yin and Yang of cellular nutrient sensing and growth control”—opposing signalling pathways that arose very early during eukaryotic evolution and that remain the underlying regulators of cellular growth control even in the presence of the additional tier of growth factor signalling that evolved with multicellularity. mTORC1 integrates amino acid availability through a sophisticated lysosomal sensing machinery involving the Rag GTPases, the Ragulator complex, and v-ATPase, with leucine and arginine serving as the best-characterised activating signals, while glutamine promotes mTORC1 activation via ADP-ribosylation factor 1 (Arf1) independently of the Rag GTPases [65]. In the cardiovascular system, Sciarretta et al. (2018, 2022) in Circulation Research and Cardiovascular Research demonstrated that mTORC1 is indispensable for embryonic cardiovascular development and for the development of adaptive cardiac hypertrophy in response to mechanical overload, yet persistent and deregulated mTORC1 activation is detrimental during stress and contributes to the development and progression of cardiac remodelling and metabolic cardiomyopathies [66]. The molecular basis of the AMPK-mTOR antagonism involves multiple reciprocal inhibitory interactions: AMPK directly phosphorylates Raptor and TSC2 to inhibit mTORC1, while mTORC1 activation suppresses AMPK through S6K1-mediated phosphorylation; SIRT1 deacetylates and activates Liver kinase B1 (LKB1) (the upstream kinase of AMPK), reinforcing nutrient deprivation signalling, while mTOR-driven protein synthesis depletes the NAD^+^ pool and reduces sirtuin activity. These reciprocal interactions create a system with bistable properties: the cardiomyocyte tends to exist in either a predominantly catabolic (AMPK/SIRT1-dominant) or predominantly anabolic (mTOR-dominant) state, with transitions between states triggered by changes in nutrient availability, hormonal milieu, and energy demand [67].

A parallel amino acid-sensing pathway functions through GCN2 (general control nonderepressible 2), a kinase that detects amino acid deprivation by binding uncharged tRNAs and subsequently activates the integrated stress response (ISR) via Eukaryotic initiation factor 2 alpha (eIF2α) phosphorylation [68]. In cardiac tissue, GCN2 activation induces the transcription factor Activating transcription factor 4 (ATF4), which orchestrates a broad transcriptional program supporting amino acid metabolism, redox homeostasis, autophagy, and proteasomal degradation. The cardiac effects of GCN2 activation are primarily maladaptive. Lu et al. (2014) in Hypertension demonstrated that GCN2 knockout mice are protected from pressure overload-induced heart failure, despite experiencing similar degrees of hypertrophy, due to preservation of sarcoplasmic/endoplasmic reticulum Ca^2+^-ATPase 2a (SERCA2a) expression and enhanced Bcl-2-mediated resistance to apoptosis [69]. Feng et al. (2019) in Free Radical Biology and Medicine further extended these findings to diabetic cardiomyopathy, showing that GCN2 deficiency ameliorates cardiac dysfunction by reducing lipotoxicity and oxidative stress through attenuation of PPARα/γ upregulation and the eIF2α-ATF4-CHOP (CCAAT/enhancer-binding protein homologous protein) axis [70]. GCN2 also interacts with mTORC1 to promote autophagic clearance of damaged proteins and organelles, integrating signals from oxidative and endoplasmic reticulum stress to restore proteome balance. These findings position GCN2 as a nutrient-sensing pathway whose chronic activation, in contrast to the generally protective effects of AMPK, contributes to cardiac dysfunction and underscores the context-dependency of nutrient sensing in the heart.

### 3.2. Transcriptional Coordination of Cardiac Metabolic Programs

The nutrient-sensing pathways described above converge on a transcriptional regulatory network that translates metabolic signals into coordinated gene expression programs. PGC-1α (PPARγ coactivator 1α) functions as a master transcriptional coactivator that does not bind DNA directly but potentiates the activity of coupled transcription factors, including the estrogen-related receptors (ERRα, ERRγ), the nuclear respiratory factors (NRF1, NRF2/Gabpa [GA-binding protein transcription factor alpha subunit]), and the PPARs [71]. PGC-1α potentiates the transcription of 13 out of 14 TCA cycle genes, partly through ERR, NRF1, Gabpa, and Yin Yang 1 (YY1), with ERR and Gabpa playing the major role, establishing that the transcriptional control of the entire oxidative metabolic machinery is channelled through a remarkably small number of coupled transcription factors.

ERRα occupies a particularly critical nodal position within this circuit. Ramjiawan et al. (2013) demonstrated the reciprocal arm of this circuit: PGC-1α expression is itself critically dependent upon ERRα in cardiomyocytes, with ERRα binding to an evolutionarily conserved site within the PGC-1α promoter—establishing a positive feedback loop (ERRα → PGC-1α → ERRα coactivation) that maintains the oxidative metabolic program [72]. Sakamoto et al. (2022) in Nature Communications extended this framework by showing that ERRγ cooperates with the cardiogenic factor GATA4 to orchestrate cardiomyocyte maturation: ERRγ occupies many cardiomyocyte enhancers and super-enhancers, often co-localising with GATA4, and the two factors cooperatively activate transcription of targets involved in contractile function, whereas ERRγ-mediated control of metabolic genes occurs independently of GATA4—both mechanisms requiring PGC-1α [73]. A disease-causing GATA4 mutation diminishes PGC-1α/ERR/GATA4 cooperativity, and ERR target genes are downregulated in human heart failure samples, suggesting that dysregulation of this circuitry contributes to both congenital and acquired forms of heart failure.

The PGC-1α/ERR/PPAR axis does not operate in isolation but is modulated by a set of recently uncovered transcriptional coregulators that fine-tune its activity in response to physiological and pathological demands. RIP140 (receptor interacting protein 140, encoded by Nrip1) functions as a transcriptional corepressor that directly antagonises PGC-1α at shared genomic targets. Yamamoto et al. (2023) in the Journal of Clinical Investigation demonstrated that RIP140 deficiency in cardiomyocytes activates genomic enhancers enriched in ERR and Myocyte enhancer factor 2 (MEF2) binding motifs, resulting in increased expression of a broad array of genes involved in mitochondrial energy metabolism and contractile function—and that cardiomyocyte-specific RIP140-deficient mice are protected against the development of heart failure caused by pressure overload combined with myocardial infarction [74].

The transcriptional output of these regulatory circuits is ultimately determined by the chromatin machinery that controls access to metabolic gene promoters and enhancers. BRD4, the most extensively studied member of the bromodomain and extra-terminal domain (BET) family, functions as an epigenetic reader that recognizes acetylated histone marks and recruits the positive transcription elongation factor b (P-TEFb) to drive transcriptional elongation [75]. In the context of cardiac metabolism, BRD4 occupies a dual role: Martin et al. (2020) in Molecular and Cellular Biology demonstrated that BRD4 is differentially recruited to promoters and super-enhancers of hypertrophic genes in a G protein-coupled receptor (GPCR)-specific manner, with protein kinase A required for α-adrenergic stimulation of BRD4 chromatin occupancy [76]. In diabetic cardiomyopathy, BRD4 expression is significantly increased, and BRD4 upregulation inhibits PTEN-induced kinase 1 (PINK1)/Parkin-mediated mitophagy, resulting in accumulation of damaged mitochondria—an effect reversed by the BET inhibitor JQ1, which rewires BRD4-driven transcription and derepresses PINK1. Mu et al. (2020) identified a novel post-translational modification of BRD4-poly(ADP-ribosyl)ation by PARP1 that facilitates BRD4 binding to transcription start sites of hypertrophic genes, establishing a direct link between NAD^+^-consuming processes (PARP1 activity) and BRD4-dependent transcriptional activation [77]. This connection is particularly significant because it creates a metabolic–epigenetic circuit: NAD^+^ depletion (as occurs in heart failure and nutrient excess) simultaneously reduces sirtuin-mediated deacetylation (increasing histone acetylation marks that recruit BRD4) and activates PARP1-mediated BRD4 PARylation (enhancing BRD4 chromatin occupancy)—a convergent mechanism that shifts the transcriptional program from oxidative metabolism toward hypertrophic and fibrotic gene expression.

The Mediator complex provides the final bridge between these transcription factor-coregulator assemblies and RNA polymerase II. MED1 (Mediator subunit 1) is essential for cardiac metabolic gene expression: cardiomyocyte-specific deletion of MED1 results in lethal dilated cardiomyopathy with downregulation of genes critical for PPAR-regulated energy metabolism, oxidative phosphorylation, and mitochondrial function, including PGC-1α, PGC-1β, and succinate dehydrogenase subunits of complex II [78].

Spitler et al. (2017) in the American Journal of Physiology demonstrated that Med1 deficiency causes significant dysregulation of genes coordinated by PGC-1α, PPARα, and ERRα, with consequent alterations in mitochondrial size, gene expression, complex activity, and electron conduction chain expression—confirming that MED1 is the critical Mediator subunit through which the PGC-1α/ERR/PPAR axis accesses the basal transcriptional machinery [79]. MED13, another Mediator subunit, adds a remarkable systemic dimension: Grueter et al. (2012) in Cell demonstrated that cardiac-specific overexpression of MED13 confers resistance to high-fat diet-induced obesity and improves systemic insulin sensitivity and glucose tolerance, while cardiac deletion of MED13 enhances obesity and exacerbates metabolic syndrome—revealing that the heart regulates systemic energy homeostasis via MED13-dependent transcription of nuclear hormone receptor target genes [80]. MED13 is itself negatively regulated by the heart-specific microRNA miR-208a, establishing a miRNA–Mediator axis through which cardiac transcriptional programs influence whole-body metabolism.

The merging of these transcriptional mechanisms is captured by the concept of “metabo-epigenetic circuitry” proposed by Pepin et al. (2025) in EMBO Molecular Medicine: chromatin-modifying enzymes function as both sensors and transducers of metabolic stress, incorporating metabolic perturbations as epigenetic modifications that regulate cardiac gene expression [81]. Flowers and Duric (2025) established that metabolic changes alter epigenetic landscapes via shifts in acetyl-CoA, NAD^+^, and α-ketoglutarate, while epigenetic dysregulation, in turn, suppresses genes involved in oxidative phosphorylation and mitochondrial biogenesis—creating bidirectional circuits in which metabolism shapes the epigenome and the epigenome shapes metabolism [82]. Although these observations strongly support the existence of metabo-epigenetic interactions, most available evidence remains associative. Direct causal links between specific dietary interventions, metabolite availability, locus-specific epigenetic remodelling, and transcriptional regulation of defined cardiac genes have yet to be conclusively demonstrated.

Laurette and Gilsbach (2026) in Nature Reviews Cardiology provided the most current framework, emphasising that advances in single-cell and cell-type-resolved epigenomic analyses have revealed the heterocellular nature of cardiac epigenetic regulation, with chromatin remodelling driven by specific modifiers, transcription factors, and chaperones that orchestrate cardiac gene expression in a cell-type-specific manner [83]. Future studies integrating tissue-specific genetic models, stable isotope-resolved metabolomics, and cell-type-resolved epigenomic profiling will be essential to establish these mechanistic relationships and distinguish direct cardiac effects from systemic metabolic adaptations.

The transcriptional coordination of cardiac metabolic programs thus emerges not as a linear cascade from nutrient signal to gene expression but as a flexible, bidirectional network in which metabolic state and transcriptional output are reciprocally coupled through the shared currency of chromatin modifications—a network whose disruption underlies the metabolic inflexibility that characterises the failing heart.

## 4. Mitochondrial Control of Diet-Dependent Cardiac Energetics

Mitochondrial dysfunction and impaired metabolic flexibility are hallmarks of cardiovascular diseases, including heart failure, diabetic cardiomyopathy, and ischemic heart disease [49,84]. Understanding how mitochondria control diet-dependent cardiac energetics is therefore essential for developing therapeutic strategies to restore metabolic homeostasis and improve cardiac outcomes.

### 4.1. Bioenergetic Efficiency and Metabolic Adaptation

Bioenergetic efficiency describes the linear relationship between myocardial oxygen consumption (MVO_2_) and cardiac work output across varying loading conditions. In healthy hearts, this relationship remains remarkably stable, with oxidative phosphorylation rates precisely matched to ATP hydrolysis rates across a wide range of work outputs. However, this efficiency is not uniform across all metabolic substrates. The heart’s ability to select optimal fuel sources, including fatty acids, glucose, lactate, ketone bodies, and amino acids, based on substrate availability, hormonal signals, and energetic demands represents a critical adaptive mechanism. Conversely, abnormally high myocardial dependence on fatty acid metabolism, as occurs during ischemia or high adrenergic states, increases cardiac oxygen consumption by 30–50% for equivalent stroke work. Metabolic adaptation encompasses the coordinated regulation of substrate utilization, oxidative capacity, and energy transfer systems through allosteric control, transcriptional reprogramming, and post-translational modifications [4]. This metabolic flexibility allows the heart to optimize ATP production efficiency under varying physiological conditions, from rest to maximal exercise, and from fed to fasted states. Conversely, loss of metabolic adaptation and reduced bioenergetic efficiency are hallmarks of cardiovascular diseases, including heart failure, diabetic cardiomyopathy, and ischemic heart disease. Several mechanisms of metabolic adaptation have been described [85,86].

The glucose–fatty acid cycle, first proposed by Randle and colleagues in 1963, describes the reciprocal relationship between carbohydrate and fatty acid metabolism [87,88]. In the fasted state, when circulating free fatty acid levels are elevated, fatty acids become the major energy source (approximately 70% of ATP production), while glucose oxidation is suppressed. This metabolic switch occurs through multiple mechanisms operating at different enzymatic steps. Fatty acid oxidation increases mitochondrial acetyl-CoA and NADH levels, which inhibit pyruvate dehydrogenase (PDH), the rate-limiting enzyme for glucose oxidation. Simultaneously, elevated citrate concentrations inhibit phosphofructokinase-1 (PFK-1), the rate-limiting enzyme of glycolysis, while increased glucose-6-phosphate inhibits hexokinase, reducing glucose uptake [89].

**Figure 6:**
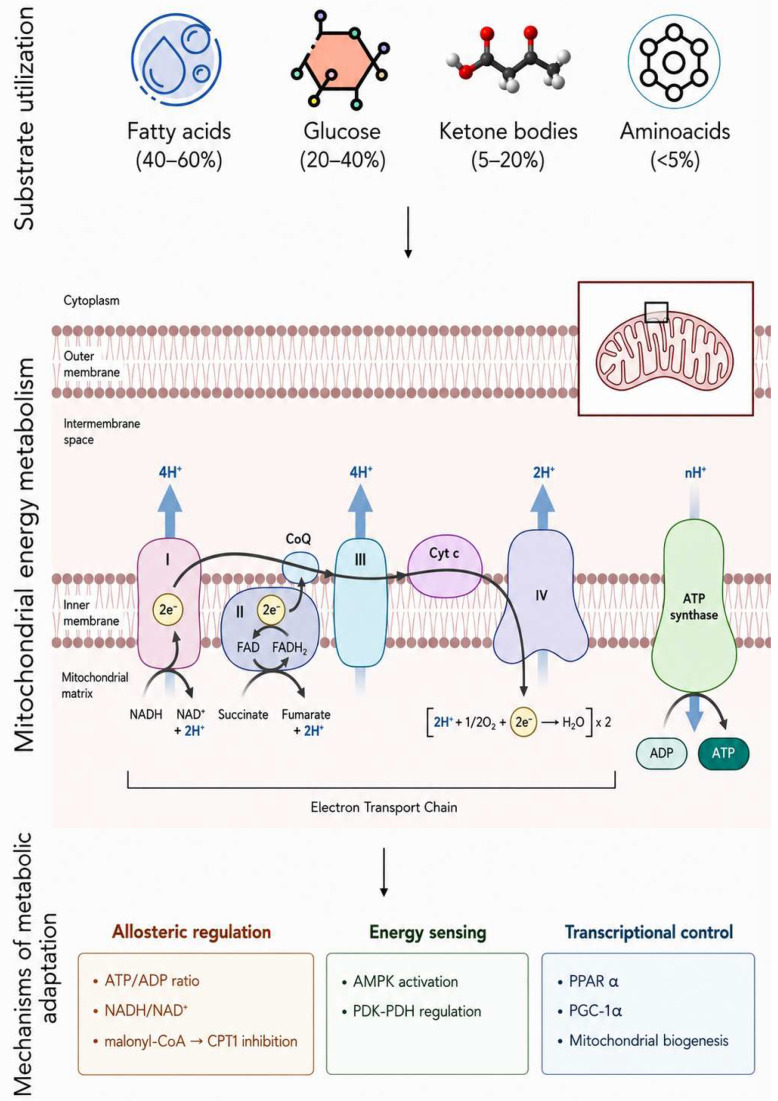
Bioenergetic efficiency and metabolic adaptation in the heart. (ADP: adenosine diphosphate; AMPK: AMP-activated protein kinase; ATP: adenosine triphosphate; CoQ: coenzyme Q (ubiquinone); CPT1: carnitine palmitoyltransferase 1; Cyt c: cytochrome c; FAD: flavin adenine dinucleotide; FADH_2_: reduced flavin adenine dinucleotide; NAD^+^: oxidized nicotinamide adenine dinucleotide; NADH: reduced nicotinamide adenine dinucleotide; PDK: pyruvate dehydrogenase kinase; PDH: pyruvate dehydrogenase; PGC-1α: peroxisome proliferator-activated receptor gamma coactivator 1-alpha; PPARα: peroxisome proliferator-activated receptor alpha. I: Complex I, NADH:ubiquinone oxidoreductase; II: Complex II, succinate dehydrogenase; III: Complex III, ubiquinol:cytochrome c oxidoreductase (cytochrome bc1 complex); IV: Complex IV, cytochrome c oxidase).

These coordinated inhibitory mechanisms effectively shut down glucose metabolism when fatty acids are abundant. Conversely, increased glucose availability and oxidation can suppress fatty acid metabolism through malonyl-CoA, which inhibits carnitine palmitoyltransferase-1 (CPT-1), the rate-limiting enzyme for mitochondrial fatty acid uptake [90]. This bidirectional regulation allows the heart to rapidly switch between fuel sources based on substrate availability and metabolic demands. However, the Randle cycle operates differently under various physiological conditions. While it effectively explains substrate competition at rest and during low-intensity exercise, it cannot fully account for substrate utilization at moderate to high exercise intensities, where additional regulatory mechanisms dominate (Figure 6). Furthermore, in advanced heart failure, excessive β-adrenergic-driven mobilization of free fatty acids may obliterate the normal diurnal glucose–fatty acid cycling, contributing to metabolic inflexibility [88].

The heart can oxidize multiple substrates including fatty acids, glucose, ketone bodies, and amino acids to sustain its high ATP demand. These substrates converge within mitochondria to generate acetyl-CoA, which fuels the tricarboxylic acid cycle and oxidative phosphorylation. Substrates differ in their oxygen efficiency, with glucose oxidation producing more ATP per oxygen molecule than fatty acid oxidation. Cardiac metabolic adaptation is regulated by allosteric control of metabolic enzymes, energy-sensing pathways such as AMPK, and transcriptional programs involving the PPARα–PGC-1 axis. These mechanisms allow the heart to dynamically adjust substrate utilization and maintain bioenergetic efficiency under changing physiological and pathological conditions.

### 4.2. Redox Signaling and Mitochondrial Stress

Mitochondria serve as the primary source of cellular reactive oxygen species (ROS), generating these highly reactive molecules as byproducts of oxidative phosphorylation [91]. Cardiac mitochondria must continuously balance ATP production with ROS generation, maintaining sufficient oxidative capacity to support contractile function while preventing oxidative damage to cellular components [92].

This delicate equilibrium is achieved through sophisticated antioxidant defense systems, redox-sensitive signaling pathways, and quality control mechanisms that monitor and respond to mitochondrial stress. The concept of mitochondrial redox signaling has evolved dramatically over the past two decades [93]. Rather than viewing ROS exclusively as damaging molecules, physiological levels of ROS are regarded as essential signaling molecules, modifying proteins and lipids to regulate mitochondrial and cardiomyocyte function. Conversely, excessive ROS production, characteristic of pathological states including heart failure, diabetic cardiomyopathy, and ischemia–reperfusion injury, leads to irreversible mitochondrial damage and contributes significantly to cardiovascular disease progression [94].

The production of ROS in diabetic hearts results from multiple sources including mitochondrial dysfunction, NADPH oxidase activation, uncoupled nitric oxide synthase, and xanthine oxidase. Prolonged increases in ROS production in diabetic cardiovascular cells activate nuclear poly(ADP-ribose) polymerase (PARP), which inhibits glyceraldehyde-3-phosphate dehydrogenase (GAPDH), shunting early glycolytic intermediates into pathogenic signaling pathways [95]. ROS and PARP also reduce sirtuin, PGC-1α, and AMPK activity, causing decreased mitochondrial biogenesis, increased ROS production, and disturbed circadian clock synchronization of glucose and lipid metabolism [96]. These interconnected pathways demonstrate how oxidative stress propagates metabolic dysfunction in diabetic hearts.

Redox signaling is tightly linked to mitochondrial stress. Under physiological conditions, low levels of mitochondrial ROS act as signaling molecules, modulating protein function and gene expression to adapt cardiac metabolism to changes in nutrient availability. However, sustained mitochondrial stress and excessive ROS production disrupt redox homeostasis, contributing to maladaptive cardiac remodeling, contractile dysfunction, and progression of cardiovascular disease. Notably, both oxidative and reductive stress can potentiate mitochondrial dysfunction. Reductive stress, characterized by an excess of electron donors, paradoxically increases ROS generation by creating an electron “traffic jam” in the ETC [97]. Dietary interventions such as caloric restriction can mitigate mitochondrial stress by reducing substrate overload, lowering ROS production, and enhancing antioxidant defenses, thereby preserving mitochondrial function and bioenergetic efficiency. Conversely, chronic overnutrition and certain dietary patterns (e.g., high-fat, omega-6-rich diets) exacerbate mitochondrial stress and redox imbalance, increasing the risk of cardiac pathology.

## 5. Diet-Induced Metabolic Stress and Cardiac Remodelling: Relevance to Cardiometabolic Disease

Heart failure represents the endpoint of multiple cardiovascular diseases and is characterized by disturbances at three levels of energy metabolism: substrate utilization, oxidative phosphorylation, and the creatine kinase reaction. The failing heart faces an energy deficit, primarily because of a decrease in mitochondrial oxidative capacity, indicated by decreased concentrations of both ATP and phosphocreatine [10]. This depletion results primarily from compromised mitochondrial oxidative metabolism, which affects the oxidation of both fatty acids and carbohydrates.

The metabolic signature of heart failure is complex and depends on the severity and type of heart failure, as well as the co-existence of common comorbidities such as obesity and type 2 diabetes. Cardiac hypertrophy and heart failure are generally characterized by a decline in fatty acid oxidation in favor of enhanced glucose uptake and glycolysis, with an uncoupling between glycolysis and glucose oxidation. Gene expression of enzymes related to fatty acid oxidation, lipid storage, and carnitine transport is downregulated in the human heart of patients with severe heart failure prior to cardiac transplantation compared with control subjects. However, the exact metabolic switch that occurs in heart failure remains controversial. While the general consensus suggests a shift from fatty acid to glucose use, clinical studies disagree as to whether fatty acid oxidation is decreased, increased, or unchanged in the failing heart [4]. This apparent contradiction can be partially explained by the type of heart failure and associated comorbidities. In heart failure associated with diabetes and obesity, myocardial fatty acid oxidation increases, while in heart failure associated with hypertension or ischemia, myocardial fatty acid oxidation decreases [98]. Irrespective of what occurs with fatty acid oxidation at the myocardial level, the hyperadrenergic state that develops in heart failure increases blood concentrations of free fatty acids, which, based on the Randle cycle, will be preferred over carbohydrates for whatever mitochondrial oxidative capacity the heart retains. With respect to glucose metabolism, the decrease in mitochondrial function in the failing heart not only limits fatty acid oxidation but also restricts glucose oxidation [99]. This decrease in glucose oxidation results from decreased activity of pyruvate dehydrogenase, the rate-limiting enzyme of glucose oxidation. The production of protons and lactate rises because of augmented glycolysis and decreased glucose oxidation in heart failure, which can decrease cardiac efficiency and is potentially detrimental [10]. The relative contribution of different fuels for mitochondrial ATP production also changes, including a decrease in glucose and amino acid oxidation and an increase in ketone oxidation.

The failing heart appears to revert toward a so-called fetal metabolic phenotype, with changes in expression and activity of metabolic enzymes consistent with this switch. These changes include altered expression of peroxisome proliferator-activated receptor (PPAR) α and PPAR γ cofactor α, regulators of mitochondrial biogenesis and fatty acid oxidation enzyme expression, processes that are decreased in the failing heart [100]. Progressive alterations of key cardiac metabolic pathways indicate impaired mitochondrial function and a metabolic switch during transition to heart failure. Comprehensive metabolic profiling has revealed that decreased carnitine shuttling and transportation precede mitochondrial dysfunction, suggesting potential therapeutic targets [101]. Across obesity, diabetes, and heart failure, several convergent mechanisms link metabolic derangement to cardiac dysfunction. Mitochondrial dysfunction emerges as a central feature, characterized by decreased oxidative capacity, increased uncoupling, and enhanced ROS production. The loss of metabolic flexibility, the inability to appropriately switch between fuel substrates in response to physiological demands, represents a critical pathophysiological feature common to all three conditions [102]. This metabolic inflexibility renders the heart vulnerable to ischemic stress and increased workload, contributing to the increased risk of developing heart failure in patients with obesity and diabetes. The accumulation of toxic lipid intermediates and the resulting lipotoxicity contribute to cardiomyocyte apoptosis, impaired calcium handling, and contractile dysfunction [103]. Oxidative stress, inflammation, and activation of maladaptive signaling pathways (including NFκB, Renin–angiotensin–aldosterone system [RAAS], and sympathetic nervous system activation) further amplify metabolic dysfunction and promote structural remodeling. These metabolic alterations, combined with neurohormonal activation, endothelial dysfunction, and altered gene expression, create a vicious cycle that perpetuates cardiac dysfunction and drives disease progression [104]. The convergence of lipotoxicity, glucotoxicity, inflammation, and impaired stress–response pathways has profound implications for disease progression and clinical vulnerability in cardiometabolic disorders. Chronic exposure of the myocardium to nutrient excess drives a gradual transition from compensated metabolic remodelling to overt structural and functional deterioration. Early adaptations, such as increased fatty acid oxidation or activation of stress–response signalling, may initially preserve cardiac output, but persistent metabolic stress ultimately exhausts these compensatory mechanisms. As mitochondrial dysfunction, oxidative damage, and impaired proteostasis accumulate, cardiomyocytes lose their capacity to maintain energy homeostasis, accelerating the progression toward diastolic dysfunction, systolic failure, and arrhythmogenesis [105].

Metabolic inflexibility represents a key determinant of vulnerability to secondary insults. Hearts exposed to chronic obesity or diabetes are less able to adapt to acute stressors such as ischemia, pressure overload, or neurohormonal activation. The inability to rapidly shift substrate utilization toward more oxygen-efficient fuels during ischemia exacerbates energetic mismatch, increases infarct size, and delays functional recovery. Similarly, heightened oxidative and inflammatory tone lowers the threshold for maladaptive remodelling in response to hypertension or volume overload, thereby amplifying the risk of heart failure development even in the absence of overt coronary artery disease. These mechanisms also help explain the heterogeneity of clinical phenotypes observed in cardiometabolic disease [106]. Individuals with similar degrees of obesity or glycemic control may exhibit markedly different cardiac outcomes depending on the balance between adaptive versus maladaptive metabolic and stress–response pathways. Genetic variation, sex-specific differences, aging, and comorbid conditions such as chronic kidney disease or sleep apnea can further modulate susceptibility by influencing mitochondrial function, inflammatory signaling, or autonomic tone [107]. Importantly, subclinical metabolic remodeling may precede detectable changes in cardiac structure or function, highlighting a prolonged window during which the myocardium is vulnerable yet potentially reversible.

From a translational perspective, these insights underscore the need to move beyond traditional hemodynamic and glycemic targets when assessing risk and guiding therapy. Interventions that restore metabolic flexibility, improve mitochondrial efficiency, dampen chronic inflammation, and normalize stress–response pathways may not only slow disease progression but also enhance cardiac resilience to acute and chronic stress. Failure to address these underlying metabolic drivers allows the persistence of a hostile myocardial environment, predisposing patients with obesity and diabetes to accelerated cardiac aging, disproportionate injury from superimposed insults, and poorer outcomes once heart failure is established.

## 6. Nutritional Modulation of Cardiac Metabolism: Implications and Open Questions

Comprehensive dietary patterns exert coordinated effects on multiple metabolic pathways that collectively influence cardiac function (Table 2). Mechanistically, the Mediterranean diet reduces atherosclerosis progression, improves endothelial function, and favourably modulates inflammation and oxidative stress through activation of AMPK-SIRT1 signaling pathways and suppression of NF-κB-mediated inflammatory responses [108].

The DASH (Dietary Approaches to Stop Hypertension) diet has shown benefits in heart failure populations, with observational data demonstrating dose-dependent mortality reduction in women with heart failure [109].

The GOURMET-HF trial demonstrated that 4 weeks of home-delivered DASH-compliant meals following heart failure hospitalization improved symptoms, functional capacity, and reduced rehospitalizations [110].

These dietary patterns share common features including high fiber content, abundant antioxidants, and emphasis on unsaturated fatty acids that collectively enhance mitochondrial efficiency and metabolic adaptability. Collectively, these findings suggest that clinically established dietary patterns influence cardiac metabolism both indirectly, by improving traditional cardiovascular risk factors, and directly, by preserving myocardial metabolic flexibility. Enhanced mitochondrial efficiency, improved substrate utilization, reduced oxidative stress, and modulation of nutrient-sensing pathways are common mechanistic features that link dietary quality to improved cardiac energetic homeostasis.

Caloric restriction and intermittent fasting represent more intensive metabolic interventions. The CALERIE trial demonstrated that 2 years of moderate (approximately 12%) caloric restriction in healthy non-obese adults significantly reduced multiple cardiometabolic risk factors including LDL cholesterol, blood pressure, C-reactive protein, and insulin resistance, with effects persisting beyond weight loss alone [111].

Animal studies reveal that caloric restriction enhances cardiac AMPK activity, reduces mitochondrial acetylation, decreases oxidative stress, increases autophagy, and improves ischemic tolerance.

In humans, caloric restriction attenuates age-related arterial stiffness, preserves left ventricular diastolic function, and reduces cardiac fibrosis [112].

Conversely, Western dietary patterns high in saturated fats and refined carbohydrates impair cardiac metabolic flexibility by disrupting nutrient-sensing pathways including AMPK, PPARα, mTOR, and SIRT1/PGC-1α, leading to mitochondrial dysfunction, oxidative stress, and maladaptive remodeling [113]. High-fat diet feeding in rodents induces cardiac insulin resistance, increases fatty acid oxidation with decreased glucose oxidation, and eventually leads to contractile dysfunction after 15–20 weeks. Omega-3 polyunsaturated fatty acids (n-3 PUFAs), particularly eicosapentaenoic acid (EPA) and docosahexaenoic acid (DHA), profoundly influence cardiac mitochondrial structure and function [114].

Ketone bodies have emerged as important alternative cardiac fuels, particularly in heart failure states. The failing heart exhibits increased ketone oxidation mediated by elevated circulating ketone levels and cardiac-autonomous upregulation of ketolytic enzymes [115].

This metabolic adaptation appears cardioprotective, as cardiac-specific deficiency in β-hydroxybutyrate dehydrogenase (BDH1) impairs cardiac function under stress conditions [116]. In humans with heart failure and reduced ejection fraction, acute infusion of β-hydroxybutyrate to achieve plasma levels of 3.3 mmol/L enhanced left ventricular ejection fraction by 8% and cardiac output by 2.0 L/min, though myocardial efficiency remained unchanged as oxygen consumption increased proportionally [117].

More recently, the randomized controlled trial of oral ketone ester treatment in heart failure patients demonstrated that 14 days of supplementation increased resting cardiac output, reduced pulmonary capillary wedge pressure at rest and during exercise, lowered NT-proBNP by 18%, increased left ventricular ejection fraction, and reduced cardiac volumes, all achieved on top of optimal medical therapy. Importantly, there exists a critical distinction between ketone supplementation strategies and ketogenic diets. While ketone esters directly elevate circulating ketone levels, ketogenic diets simultaneously increase circulating fatty acids, which may negate potential cardiac benefits [118]. Indeed, ketogenic diets consistently increase LDL cholesterol and lack randomized trial evidence for cardiovascular event reduction. Branched-chain amino acids (BCAAs, leucine, isoleucine, valine) have emerged as biomarkers and potential mediators of cardiometabolic disease. Elevated BCAA levels are associated with insulin resistance, type 2 diabetes, obesity, and incident cardiovascular events [119].

In the PREMIER lifestyle trial, increases in BCAA levels over 6 months were associated with increases in insulin resistance (Homeostatic model assessment of insulin resistance-HOMA-IR), inflammation (Glycoprotein acetylation-GlycA), apolipoprotein B, and Very-low-density lipoprotein (VLDL) cholesterol. Mechanistically, chronic BCAA accumulation enhances cardiac fatty acid oxidation through activation of the GCN2/ATF6/PPARα pathway, exacerbates lipid peroxidation toxicity, and worsens myocardial vulnerability to ischemia–reperfusion injury [39].

In genetic mouse models with defective BCAA catabolism, chronic BCAA accumulation increases cardiac fatty acid oxidation and sensitizes the heart to ischemia–reperfusion injury, effects reversed by PPARα silencing [1]. Additionally, BCAAs promote cardiovascular disease through activation of mTOR/SREBP-1/betatrophin pathways that enhance triglyceride metabolism [120].

Importantly, circulating BCAA levels are modifiable through dietary interventions, including caloric restriction and Mediterranean diet patterns, suggesting potential therapeutic targets. However, the complex bidirectional relationship between BCAAs and metabolic disease, where early insulin resistance elevates BCAAs, which then aggravate metabolic dysfunction, complicates intervention strategies.

Specific micronutrient deficiencies are common in heart failure and may contribute to metabolic dysfunction. Iron deficiency, present in up to 50% of heart failure patients, impairs mitochondrial function and exercise capacity. Intravenous (but not oral) iron supplementation improves functional status, exercise capacity, and quality of life in iron-deficient heart failure patients [121]. Thiamine deficiency, particularly common with diuretic use, can cause reversible cardiomyopathy (wet beriberi), and supplementation has improved ejection fraction in small trials [122].

Coenzyme Q10, an essential component of the mitochondrial electron transport chain with antioxidant properties, demonstrated reduced all-cause mortality in heart failure patients (RR 0.69, 95% CI 0.50–0.96) in meta-analyses, though evidence certainty remains low [123].

Vitamin D, magnesium, selenium, and zinc deficiencies are prevalent in heart failure, but supplementation trials have shown inconsistent benefits, highlighting the complexity of micronutrient–metabolism interactions [124].

The gut microbiome serves as a critical intermediary linking diet to cardiac metabolism through production of bioactive metabolites. Short-chain fatty acids (SCFAs), acetate, propionate, and butyrate, produced by bacterial fermentation of dietary fiber, act as histone deacetylase inhibitors and signaling molecules that modulate inflammation, blood pressure, and cardiac remodeling. Mediterranean and plant-based diets, rich in fiber precursors for SCFA production, favorably alter gut microbiome composition and metabolite profiles [125]. Machine learning models incorporating gut microbiome data can predict individual metabolic responses to complex meals with high accuracy, and microbiome-guided dietary interventions improve glycemic control more effectively than standard nutritionist recommendations [126].

These findings support the potential for gut microbiome-based precision nutrition approaches in heart failure management, though clinical validation remains needed.

Nutrients function as epigenetic modulators, influencing gene expression through DNA methylation, histone modifications, and non-coding RNA regulation. Cardioprotective dietary patterns induce favorable epigenetic remodeling involving coordinated activation of AMPK-SIRT1 signaling, suppression of mTOR overactivation, and modulation of NF-κB and Nrf2 pathways. These molecular changes enhance mitochondrial biogenesis, improve redox balance, reduce inflammation, and promote metabolic adaptability. Specific nutrients demonstrate distinct epigenetic effects. Omega-3 PUFAs modulate microRNA expression, thereby impacting lipid metabolism and inflammatory responses [127]. Polyphenols from fruits, vegetables, and olive oil influence histone acetylation and DNA methylation patterns associated with cardiovascular protection. Caloric restriction induces widespread epigenetic changes, including reduced histone acetylation and altered expression of longevity-associated genes. These epigenetic modifications can be long-lasting and potentially transmissible across generations through germline DNA and mitochondrial modifications, supporting early-life nutritional interventions for cardiovascular disease prevention. The integration of nutrigenomics with clinical practice promises to identify individuals most likely to benefit from specific dietary interventions based on genetic polymorphisms affecting nutrient metabolism and cardiovascular risk.

**Table 2:** Evidence from human and translational studies on nutritional modulation of cardiac metabolism.

	Study	Population	Key Findings	Ref.
**Mediterranean diet**	PREDIMED	High CV risk adults	29% reduction in major adverse CV events; 42% reduction in stroke over 4.8 years	[128]
	CORDIOPREV	Established coronary disease	26% reduction in major CV events over 7 years vs. low-fat diet	[129]
	Lyon Diet Heart Study	Post-MI patients	76% reduction in fatal CVD (HR 0.24); 73% reduction in non-fatal MI + CVD death over 46 months	[130]
**DASH diet**	Women’s Health Initiative	Women with HF	Dose-dependent mortality reduction with greater adherence (most adherent HR 0.84)	[131]
	GOURMET-HF	Post-HF hospitalization ≥ 65 yrs	4 weeks of home-delivered DASH meals improved symptoms, functional capacity, reduced rehospitalizations	[110]
**DASH/MED/AHEI**	Meta-analysis	General population	25% lower HF risk with highest vs. lowest adherence; linear dose–response relationship	[132]
**Caloric Restriction**	CALERIE	Healthy non-obese adults (21–50 years)	12% calorie reduction over 2 years: significant reductions in LDL, blood pressure, CRP, insulin resistance, metabolic syndrome score	[111]
	Meta-analysis	Adults	1–4 weeks: SBP −5.5 mmHg, DBP −2.9 mmHg; 1.5–6 months: HR −4.4 bpm, VO_2_peak +1.8 mL/kg/min	[133]
	Animal/Human studies	Various	Enhanced cardiac AMPK activity, reduced mitochondrial acetylation, decreased oxidative stress, increased autophagy, improved ischemic tolerance	[134]
**Ketogenic Diet**	Various studies	HF models	Minimal cardiac benefit; increases LDL cholesterol; concurrent fatty acid elevation may negate ketone benefits	[135]

Despite substantial progress, significant methodological limitations constrain translation of nutritional metabolic research into clinical practice. Most dietary intervention trials in heart failure populations are small, short-duration studies with surrogate endpoints rather than hard clinical outcomes [136].

The CORDIOPREV trial represents a notable exception, but even this landmark study had relatively low certainty of evidence for Mediterranean diet effects on heart failure incidence. Randomized controlled trial data are lacking for several common interventions including intermittent fasting, ketogenic diets, and specific dietary patterns in heart failure with preserved ejection fraction.

Inherent challenges in nutrition research include difficulty conducting double-blind randomized trials, high variability in dietary adherence, complex interactions between multiple nutrients, and long latency periods between dietary exposures and cardiovascular outcomes [137].

Observational studies are subject to residual confounding by socioeconomic factors, health behaviors, and access to healthcare. Furthermore, most studies have examined dietary effects on heart failure as a composite outcome without distinguishing between heart failure with reduced versus preserved ejection fraction, which may have distinct metabolic phenotypes requiring different nutritional strategies [138].

The complex relationship between BCAAs and cardiovascular disease illustrates the challenge of distinguishing biomarkers from causal mediators. While elevated BCAAs associate with cardiovascular risk and mechanistic studies demonstrate pathogenic effects, whether BCAA-lowering interventions will improve cardiovascular outcomes remains unproven [139].

Similarly, the optimal balance between different fatty acid classes (saturated, monounsaturated, omega-3, omega-6) for cardiac metabolic health requires more precise definition across different disease states. The role of circadian rhythm and chrononutrition in cardiac metabolism represents an emerging area with limited human data. Time-restricted eating and meal timing may influence cardiac metabolic efficiency and substrate utilization, but optimal timing strategies for cardiovascular disease prevention and heart failure management remain undefined [140].

Fundamental questions remain regarding the precise mechanisms by which dietary interventions modulate cardiac metabolism. While ketone bodies clearly increase in heart failure and acute ketone administration improves hemodynamics, the mechanisms underlying these benefits, whether related to enhanced ATP production, signaling effects, epigenetic modifications, or extra-cardiac actions, remain incompletely defined. The relative contributions of different ketone bodies (β-hydroxybutyrate versus acetoacetate) and optimal dosing strategies require further investigation. Although ketone bodies have been proposed as “thrifty fuels” that enhance cardiac efficiency, acute infusion studies demonstrate that while ketones increase cardiac output and ejection fraction, myocardial oxygen consumption increases proportionally, leaving efficiency unchanged [141].

Whether chronic ketone elevation through supplementation produces different efficiency effects than acute infusion remains unknown. Furthermore, the pleiotropic signalling properties of β-hydroxybutyrate, including HDAC inhibition, modulation of inflammatory pathways, and effects on oxidative stress, may contribute substantially to clinical benefits independent of fuel provision, but the relative importance of these mechanisms requires clarification. The complex relationship between BCAAs and cardiovascular disease illustrates the challenge of distinguishing biomarkers from causal mediators. While elevated BCAAs associate with cardiovascular risk and mechanistic studies demonstrate pathogenic effects through activation of mTOR/SREBP-1/betatrophin pathways and enhancement of cardiac fatty acid oxidation via GCN2/ATF6/PPARα signaling, whether BCAA-lowering interventions will improve cardiovascular outcomes remains unproven [142].

The bidirectional nature of BCAA dysregulation, where insulin resistance elevates circulating BCAAs, which then aggravate metabolic dysfunction, complicates intervention strategies. Moreover, recent evidence suggests divergent effects of individual BCAAs (valine versus leucine and isoleucine) on lipid metabolism, indicating that therapeutic approaches may need to target specific amino acids rather than total BCAA levels [143]. The role of BCAA catabolic defects in heart failure pathogenesis has been established through genetic models showing that impaired BCAA oxidation promotes cardiac dysfunction, oxidative stress, and metabolic disturbance, yet optimal strategies to restore BCAA catabolism, whether through dietary manipulation, pharmacological enhancement of branched-chain α-keto acid dehydrogenase activity, or transcriptional modulation, require systematic investigation in human trials [144]. Similarly, the optimal balance between different fatty acid classes (saturated, monounsaturated, omega-3, omega-6) for cardiac metabolic health requires more precise definition across different disease states. While omega-3 PUFAs clearly improve mitochondrial function and reduce cardiovascular events in some populations, recent trials have identified increased atrial fibrillation risk with high-dose supplementation, suggesting dose-dependent and potentially phenotype-specific effects that remain poorly characterized [145].

The mechanisms by which omega-3 PUFAs alter mitochondrial membrane composition, enhance respiratory capacity, and delay calcium-induced permeability transition are established, but how these molecular changes translate into clinical outcomes across diverse heart failure phenotypes requires further elucidation. The metabolic distinctions between heart failure with preserved ejection fraction (HFpEF) and heart failure with reduced ejection fraction (HFrEF) represent a critical knowledge gap with direct implications for nutritional interventions. Despite marked obesity and diabetes in HFpEF patients, myocardial metabolomics reveal paradoxically lower fatty acid metabolites, ketones, tricarboxylic acid cycle intermediates, and BCAA metabolites compared to HFrEF, suggesting profound fuel inflexibility rather than substrate overload [146].

HFpEF myocardium exhibits higher pyruvate but lower succinate and fumarate, with elevated non-branched-chain and branched-chain amino acids yet decreased downstream metabolites and suppressed genes controlling BCAA metabolism. These metabolic signatures are not detectable in plasma, highlighting the inadequacy of circulating biomarkers for assessing myocardial metabolism. In contrast, HFrEF demonstrates increased ketone oxidation as an adaptive response, with cardiac-specific deficiency in ketolytic enzymes impairing function under stress. The distinct calcium handling properties, diminished calcium release in HFrEF versus enhanced release in HFpEF cardiomyocytes, further suggest fundamentally different cellular energetic demands [147]. Whether nutritional interventions should differ between these phenotypes, and how to optimize substrate provision for the fuel-inflexible HFpEF myocardium, remains undefined. Most dietary intervention trials have not stratified by ejection fraction phenotype, limiting phenotype-specific therapeutic guidance. The role of circadian rhythm and chrononutrition in cardiac metabolism represents an emerging area with limited human data but substantial mechanistic plausibility. The circadian system regulates cardiac metabolism, vascular function, and cardiac performance through 24 h rhythms in heart rate, blood pressure, cholesterol synthesis, inflammatory cytokine expression, and autonomic output [148]. Meal timing acts as a zeitgeber for peripheral circadian clocks in metabolic organs, and misalignment between meal timing and the central clock can induce internal circadian disruption. Epidemiologic evidence links irregular eating patterns, late-night eating, and prolonged eating windows with greater risk of obesity, type 2 diabetes, and cardiovascular disease. Having a later first meal (after 9 AM) and last meal (after 9 PM) is associated with higher cardiovascular risk, especially in women [149].

Time-restricted feeding reinforces feeding–fasting rhythms without caloric reduction and ameliorates metabolic disorders including obesity and cardiac dysfunction through optimizing gene expression related to normal metabolic function. However, optimal timing strategies for cardiovascular disease prevention and heart failure management remain undefined. Critical questions include: What is the optimal eating window duration? How should meal timing be individualized based on chronotype? Does the timing of specific macronutrients (e.g., carbohydrates earlier versus later in the day) differentially affect cardiac metabolism? These questions require investigation through controlled trials with cardiac metabolic phenotyping. The gut microbiome represents a critical intermediary linking diet to cardiac metabolism, yet mechanistic understanding remains incomplete. SCFAs produced by bacterial fermentation of dietary fiber act as histone deacetylase inhibitors and signaling molecules that modulate inflammation, blood pressure, and cardiac remodeling, but the relative contributions of individual SCFAs (acetate, propionate, butyrate) to cardiovascular protection, their tissue-specific effects, and optimal strategies to enhance their production require clarification [150]. Conversely, TMAO produced from gut bacterial metabolism of choline and L-carnitine associates with cardiovascular risk, but whether TMAO is a causal mediator or simply a biomarker of red meat consumption and unfavorable dietary patterns remains debated. Interventional studies targeting TMAO production through dietary modification or microbial manipulation are needed to establish causality. Furthermore, the gut microbiome influences BCAA metabolism, with certain bacterial species capable of both producing and consuming BCAAs, yet how microbiome-mediated BCAA regulation affects cardiac metabolism is poorly understood [151].

Machine learning models incorporating gut microbiome data can predict individual metabolic responses to meals, suggesting potential for microbiome-guided precision nutrition, but clinical validation in cardiovascular populations is lacking [152]. The temporal dynamics of microbiome changes in response to dietary interventions, the stability of these changes after intervention cessation, and the identification of keystone species critical for cardiovascular protection represent important research priorities.

An emerging frontier in cardiovascular nutrition is the transition from population-based dietary recommendations toward precision nutrition strategies tailored to individual metabolic characteristics. Advances in metabolomics, lipidomics, and other multi-omics technologies now allow comprehensive characterisation of metabolic phenotypes that may predict individual responses to specific dietary interventions. Similarly, growing knowledge of the gut microbiome has highlighted the potential for microbiome-guided nutritional approaches, in which dietary recommendations are tailored to microbial composition and metabolite production. Beyond biological profiling, artificial intelligence and machine-learning algorithms are increasingly being integrated with clinical, nutritional, and omics data to identify personalised dietary strategies and improve prediction of cardiometabolic risk. Although these approaches remain largely investigational, they represent a promising opportunity to move beyond the traditional “one-size-fits-all” paradigm of nutritional counselling. Future clinical studies integrating advanced metabolic phenotyping, microbiome analysis, and digital decision-support tools will be essential to establish whether precision nutrition can effectively optimise myocardial metabolism and improve cardiovascular outcomes. Although substantial progress has been achieved in understanding diet-associated regulation of cardiac metabolism, several translational challenges remain unresolved. Clinical studies differ substantially in dietary protocols, duration of intervention, patient characteristics, and metabolic endpoints, limiting direct comparison across investigations. Furthermore, relatively few intervention studies incorporate detailed cardiac metabolic phenotyping, making it difficult to establish whether improvements in cardiovascular outcomes are directly mediated by myocardial metabolic remodeling or primarily reflect systemic metabolic benefits. Addressing these limitations will be essential for translating mechanistic discoveries into precision nutritional strategies for cardiovascular disease prevention and treatment. Overall, the strongest clinical evidence currently supports the cardiovascular benefits of healthy dietary patterns, whereas direct mechanistic validation of their myocardial metabolic effects in humans remains an important unmet research need.

## 7. Current Limitations, Knowledge Gaps, and Future Research Directions

Although considerable progress has been made in elucidating the molecular links between nutrition and cardiac metabolism, several important limitations hinder the translation of these findings into clinical practice. First, much of the mechanistic evidence derives from in vitro experiments and animal models, which afford valuable biological insights but do not fully recapitulate the complexity of human cardiovascular disease. Second, human studies are highly heterogeneous with respect to dietary interventions, follow-up duration, patient characteristics, comorbidities, and clinical endpoints, making direct comparisons across studies difficult and limiting the generalizability of their findings. Furthermore, dietary patterns represent complex combinations of nutrients and bioactive compounds, rendering it difficult to attribute specific metabolic effects to individual dietary components. The influence of additional modifiers—including age, sex, genetic background, metabolic status, medical therapies, physical activity, and gut microbiota composition—increases the complexity of the interpretation of the available evidence. Finally, most clinical investigations rely on systemic metabolic biomarkers, whereas direct assessment of myocardial substrate utilisation and metabolic flexibility in humans remains limited by the availability of advanced imaging techniques and validated metabolic biomarkers. These limitations emphasise several important knowledge gaps that should be addressed by future research. A better understanding of the temporal sequence linking dietary exposures to myocardial metabolic remodelling, the relative contributions of different nutrient-sensing pathways across distinct cardiovascular phenotypes, and the interactions among circadian biology, the gut microbiota, and cardiac metabolism remains required. Future investigations should integrate longitudinal clinical studies with advanced metabolic phenotyping, multi-omics technologies, and functional cardiac imaging to identify robust biomarkers of metabolic flexibility and myocardial energetic remodelling. Such approaches will facilitate the development of precision nutrition strategies tailored to individual metabolic profiles and cardiovascular phenotypes, ultimately improving the prevention and management of cardiovascular disease.

## 8. Conclusions

The traditional perspective of diet as solely a source of energy and nutrients has shifted toward a more comprehensive recognition of nutrition as a critical determinant of cardiovascular health. Evidence presented in this review demonstrates that dietary habits have significant and lasting effects on myocardial biology, influencing cardiac adaptation to physiological demands and responses to metabolic stress. Rather than acting through isolated mechanisms, dietary exposures shape an integrated metabolic phenotype that contributes to cardiovascular resilience or susceptibility to disease.

Current research highlights that the relationship between diet and cardiac function is both dynamic and bidirectional. Nutritional patterns influence myocardial performance across the lifespan, while the metabolic state of the heart determines its ability to respond to environmental and pathological challenges. In this context, metabolic flexibility is a fundamental hallmark of cardiac health, whereas its gradual decline characterises the progression toward cardiometabolic disease and heart failure.

Despite significant advances in the field, critical questions persist regarding the mechanisms underlying individual variability in dietary responses and the long-term effects of metabolic remodelling.

Emerging evidence further suggests that baseline metabolic status—including the degree of insulin resistance, metabolic flexibility, and mitochondrial function—may substantially influence the myocardial response to dietary interventions. Consequently, identical nutritional strategies may elicit distinct molecular and functional adaptations depending on the pre-existing metabolic phenotype, highlighting the need for metabolically informed patient stratification in precision nutrition [153].

Importantly, much of the current mechanistic knowledge derives from preclinical models, whereas robust human studies combining dietary interventions with advanced metabolic imaging, multi-omics profiling, and longitudinal cardiovascular phenotyping remain relatively exiguous. Bridging this gap will be critical for establishing causal mechanisms and identifying patient-specific therapeutic targets.

Particular attention should be directed toward establishing causal links between diet-induced changes in metabolite availability, epigenetic remodeling, and the regulation of specific cardiac metabolic genes, thereby distinguishing direct myocardial mechanisms from systemic metabolic adaptations. Future research that integrates nutritional assessment with advanced phenotyping will be essential for identifying patient-specific metabolic signatures and refining both preventive and therapeutic strategies.

The reviewed evidence suggests that the biological effects of individual nutrients cannot be interpreted in isolation, as their metabolic consequences depend on overall dietary context, nutrient interactions, and the individual’s metabolic state. Rather than establishing universal therapeutic thresholds for single nutrients, future nutritional strategies should emphasise holistic dietary patterns tailored to metabolic flexibility, disease stage, and energetic requirements, thereby supporting a precision nutrition approach.

**Figure 7:**
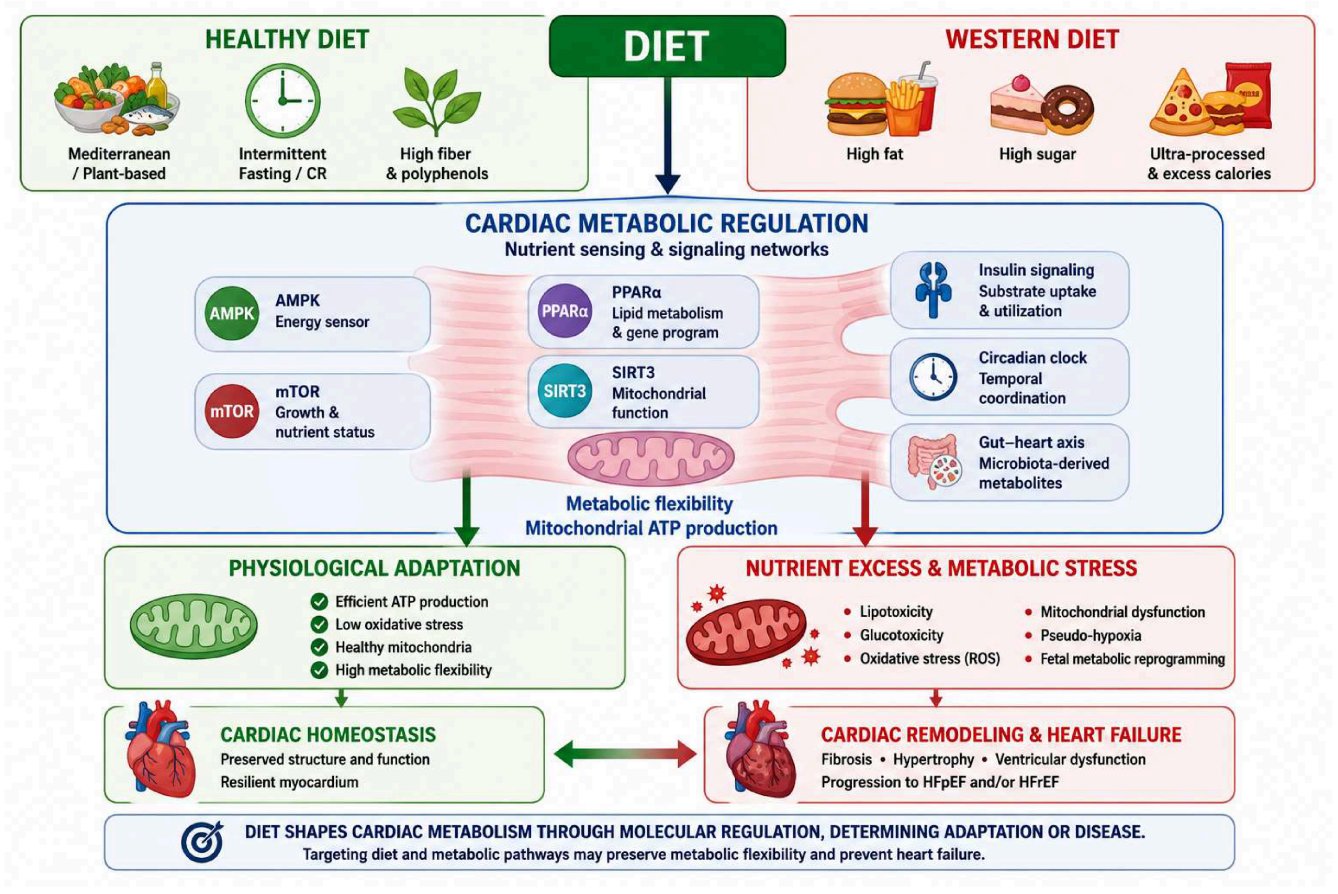
Graphical summary (AMPK: AMP-activated protein kinase; ATP: adenosine triphosphate; CR: caloric restriction; HFpEF: heart failure with preserved ejection fraction; HFrEF: heart failure with reduced ejection fraction; IF: intermittent fasting; mTOR: mechanistic target of rapamycin; PPARα: peroxisome proliferator-activated receptor alpha; ROS: reactive oxygen species; SIRT3: sirtuin 3).

The increasing integration of nutritional science and cardiovascular medicine supports a paradigm in which diet is regarded not only as a modifiable risk factor but also as a potent regulator of cardiac phenotype (Figure 7). Applying this knowledge to develop effective and personalised nutritional interventions may offer one of the most promising opportunities for improving cardiovascular health in the coming decades.

## Figures and Tables

**Table 1:** Major dietary components regulating cardiac metabolism: molecular targets, metabolic pathways, and cardiovascular consequences (AGE: advanced glycation end-products; ATF6: activating transcription factor 6; BCAA, branched-chain amino acid; BMAL1: brain and muscle ARNT-like protein 1; CLOCK: circadian locomotor output cycles kaput; GCN2: general control nonderepressible 2; HDAC: histone deacetylase; HF: heart failure; HFpEF: heart failure with preserved ejection fraction; IL-6: interleukin-6; LDL: low-density lipoprotein; mTOR: mechanistic target of rapamycin; NF-κB: nuclear factor kappa-light-chain-enhancer of activated B cells; NO: nitric oxide; O-GlcNAc: O-linked β-N-acetylglucosamine; PGC-1α: peroxisome proliferator-activated receptor gamma coactivator 1-alpha; PPARα: peroxisome proliferator-activated receptor alpha; SCFA: short-chain fatty acid; SIRT3: sirtuin 3).

Major Dietary Component/Intervention	Principal Molecular Targets	Main Metabolic Pathways Affected	Cardiovascular Outcomes
**Western diet**	CD36, PPARα, NF-κB, IL-6, insulin signaling	Increased fatty acid uptake, insulin resistance, lipotoxicity, glucotoxicity, mitochondrial dysfunction, impaired metabolic flexibility	Endothelial dysfunction, myocardial fibrosis, adverse remodeling, HFpEF
**Mediterranean diet**	AMPK, SIRT3, PPARα–PGC-1α, endothelial NO signaling	Enhanced mitochondrial function, preserved metabolic flexibility, reduced oxidative stress and inflammation	Reduced cardiovascular risk, improved myocardial energetic efficiency, lower HF incidence
**Plant-based/high-fiber diet**	Gut microbiota, SCFAs, HDAC inhibition	Increased SCFA production, improved endothelial function, attenuation of inflammatory signaling	Reduced vascular dysfunction and adverse cardiac remodeling
**High fructose intake**	Hexosamine biosynthetic pathway, O-GlcNAcylation, AGE formation	Unregulated glycolytic flux, glucotoxicity, oxidative stress, lipid accumulation	Cardiomyocyte dysfunction and adverse remodeling
**Caloric restriction**	AMPK, SIRT3, mTOR, autophagy	Enhanced mitochondrial efficiency, preserved insulin sensitivity, increased metabolic flexibility	Reduced hypertrophy, improved myocardial energetics and ischemic tolerance
**Intermittent fasting/time-restricted eating**	AMPK, BMAL1/CLOCK, ketone metabolism	Physiological substrate switching, circadian metabolic alignment, enhanced mitochondrial function	Improved cardiac performance and metabolic efficiency
**Ketogenic diet**	PPARα, ketone metabolism	Increased ketone utilization, reduced glucose oxidation	Limited direct cardiac benefit; possible increase in LDL cholesterol despite enhanced ketone availability
**Excess branched-chain amino acids (BCAAs)**	GCN2–ATF6, PPARα, mTOR	Reduced glucose oxidation, increased fatty acid oxidation, metabolic inflexibility	Increased susceptibility to ischemic injury and progression of cardiac dysfunction

## Data Availability

No new data were created or analyzed in this study. Data sharing is not applicable to this article.

## References

[1] Honka H., Solis-Herrera C., Triplitt C., Norton L., Butler J., DeFronzo R.A. (2021). Therapeutic Manipulation of Myocardial Metabolism: JACC State-of-the-Art Review. J. Am. Coll. Cardiol..

[2] Glatz J.F.C., Heather L.C., Larsen T.S., Nabben M., Luiken J.J.F.P. (2026). The Metabolic Balancing Act between Fatty Acid and Glucose Substrate Use for Optimal Myocardial Function: Key Role for Membrane Substrate Transporters. Am. J. Physiol. Heart Circ. Physiol..

[3] Taegtmeyer H., Young M.E., Lopaschuk G.D., Abel E.D., Brunengraber H., Darley-Usmar V., Des Rosiers C., Gerszten R., Glatz J.F., Griffin J.L. (2016). Assessing Cardiac Metabolism: A Scientific Statement from the American Heart Association. Circ. Res..

[4] Karwi Q.G., Jörg A.R., Lopaschuk G.D. (2019). Allosteric, Transcriptional and Post-Translational Control of Mitochondrial Energy Metabolism. Biochem. J..

[5] Hopkins T.A., Dyck J.R.B., Lopaschuk G.D. (2003). AMP-Activated Protein Kinase Regulation of Fatty Acid Oxidation in the Ischaemic Heart. Biochem. Soc. Trans..

[6] Houten S.M., Chegary M., Te Brinke H., Wijnen W.J., Glatz J.F.C., Luiken J.J.F.P., Wijburg F.A., Wanders R.J.A. (2009). Pyruvate Dehydrogenase Kinase 4 Expression Is Synergistically Induced by AMP-Activated Protein Kinase and Fatty Acids. Cell. Mol. Life Sci..

[7] Steinbusch L.K.M., Schwenk R.W., Ouwens D.M., Diamant M., Glatz J.F.C., Luiken J.J.F.P. (2011). Subcellular Trafficking of the Substrate Transporters GLUT4 and CD36 in Cardiomyocytes. Cell. Mol. Life Sci..

[8] Luiken J.J.F.P., Nabben M., Neumann D., Glatz J.F.C. (2020). Understanding the Distinct Subcellular Trafficking of CD36 and GLUT4 during the Development of Myocardial Insulin Resistance. Biochim. Biophys. Acta Mol. Basis Dis..

[9] Simsek Papur O., Glatz J.F.C., Luiken J.J.F.P. (2024). Protein Kinase-D1 and Downstream Signaling Mechanisms Involved in GLUT4 Translocation in Cardiac Muscle. Biochim. Biophys. Acta Mol. Cell Res..

[10] Lopaschuk G.D., Karwi Q.G., Tian R., Wende A.R., Abel E.D. (2021). Cardiac Energy Metabolism in Heart Failure. Circ. Res..

[11] Lichtenstein A.H., Khera A., Anderson C.A.M., Appel L.J., DeSilva D.M., Gardner C., Hu F.B., Jones D.W., Petersen K.S., American Heart Association (2026). 2026 Dietary Guidance to Improve Cardiovascular Health: A Scientific Statement from the American Heart Association. Circulation.

[12] Williams K.A., Aggarwal M., Agustina R., Dastmalchi L.N., Ferdinand K.C., Lohr N.L., Panjrath G.S. (2026). Nutrition and Front-of-Package Food Labeling as a Catalyst for Cardiovascular Health: 2025 ACC Concise Clinical Guidance: A Report of the American College of Cardiology Solution Set Oversight Committee. J. Am. Coll. Cardiol..

[13] Adolph T.E., Tilg H. (2024). Western Diets and Chronic Diseases. Nat. Med..

[14] Kwiatkowski G., Bar A., Jasztal A., Chłopicki S. (2021). MRI-Based in Vivo Detection of Coronary Microvascular Dysfunction before Alterations in Cardiac Function Induced by Short-Term High-Fat Diet in Mice. Sci. Rep..

[15] Ternacle J., Wan F., Sawaki D., Surenaud M., Pini M., Mercedes R., Ernande L., Audureau E., Dubois-Rande J.-L., Adnot S. (2017). Short-Term High-Fat Diet Compromises Myocardial Function: A Radial Strain Rate Imaging Study. Eur. Heart J. Cardiovasc. Imaging.

[16] Oka S.-I., Sung E.-A., Zhai P., Schesing K.B., Bhat S., Chin A., Park J., Chung Y.-J., Shirakabe A., Yamamoto T. (2026). PPARα-NF-κB Heterodimer Mediates Obesity-Induced Diastolic Dysfunction through Autocrine Production of IL-6. J. Clin. Investig..

[17] Liu I.-F., Lin T.-C., Wang S.-C., Yen C.-H., Li C.-Y., Kuo H.-F., Hsieh C.-C., Chang C.-Y., Chang C.-R., Chen Y.-H. (2023). Long-Term Administration of Western Diet Induced Metabolic Syndrome in Mice and Causes Cardiac Microvascular Dysfunction, Cardiomyocyte Mitochondrial Damage, and Cardiac Remodeling Involving Caveolae and Caveolin-1 Expression. Biol. Direct.

[18] Yamamoto T., Endo J., Kataoka M., Matsuhashi T., Katsumata Y., Shirakawa K., Yoshida N., Isobe S., Moriyama H., Goto S. (2018). Decrease in Membrane Phospholipids Unsaturation Correlates with Myocardial Diastolic Dysfunction. PLoS ONE.

[19] Ashraf S., Yilmaz G., Chen X., Harmancey R. (2020). Dietary Fat and Sugar Differentially Affect β-Adrenergic Stimulation of Cardiac ERK and AKT Pathways in C57BL/6 Male Mice Subjected to High-Calorie Feeding. J. Nutr..

[20] Delbridge L.M.D., Benson V.L., Ritchie R.H., Mellor K.M. (2016). Diabetic Cardiomyopathy: The Case for a Role of Fructose in Disease Etiology. Diabetes.

[21] Malik V.S., Hu F.B. (2015). Fructose and Cardiometabolic Health: What the Evidence from Sugar-Sweetened Beverages Tells Us. J. Am. Coll. Cardiol..

[22] Feng S., Jiang Y., Jiang J., Bian H., Zhu R. (2026). Effects of Diet-Modulated Gut Microbiota and Microbial Metabolites in Atherosclerosis. Biomed. Pharmacother..

[23] Kondapalli N., Katari V., Dalal K.K., Paruchuri S., Thodeti C.K. (2025). Microbiota in Gut-Heart Axis: Metabolites and Mechanisms in Cardiovascular Disease. Compr. Physiol..

[24] He Z., Liu B., Gong A., Jia X. (2026). The Role of Western Diet and Gut Microbiota in the Pathogenesis of Cardiovascular Diseases. Front. Microbiol..

[25] Arumugam T.V., Alli-Shaik A., Liehn E.A., Selvaraji S., Poh L., Rajeev V., Cho Y., Cho Y., Kim J., Kim J. (2023). Multiomics Analyses Reveal Dynamic Bioenergetic Pathways and Functional Remodeling of the Heart during Intermittent Fasting. eLife.

[26] Søndergaard A.M., Kjærulff M.L.G., Gormsen L.C., Søndergaard E. (2026). Cardiometabolic Effects of Time-Restricted Eating. Am. J. Physiol. Cell Physiol..

[27] Moellmann J., Mann P.A., Kappel B.A., Kahles F., Klinkhammer B.M., Boor P., Kramann R., Ghesquiere B., Lebherz C., Marx N. (2022). The Sodium-Glucose Co-Transporter-2 Inhibitor Ertugliflozin Modifies the Signature of Cardiac Substrate Metabolism and Reduces Cardiac mTOR Signalling, Endoplasmic Reticulum Stress and Apoptosis. Diabetes Obes. Metab..

[28] Adeva-Andany M.M., Carneiro-Freire N., Seco-Filgueira M., Fernández-Fernández C., Mouriño-Bayolo D. (2019). Mitochondrial β-Oxidation of Saturated Fatty Acids in Humans. Mitochondrion.

[29] van Weeghel M., Abdurrachim D., Nederlof R., Argmann C.A., Houtkooper R.H., Hagen J., Nabben M., Denis S., Ciapaite J., Kolwicz S.C. (2018). Increased Cardiac Fatty Acid Oxidation in a Mouse Model with Decreased Malonyl-CoA Sensitivity of CPT1B. Cardiovasc. Res..

[30] Rodríguez-Rodríguez R., Baena M., Zagmutt S., Paraiso W.K., Reguera A.C., Fadó R., Casals N. (2025). International Union of Basic and Clinical Pharmacology. CXIX. Fundamental Insights and Clinical Relevance Regarding the Carnitine Palmitoyltransferase Family of Enzymes. Pharmacol. Rev..

[31] Ciccarelli M., Santulli G., Pascale V., Trimarco B., Iaccarino G. (2013). Adrenergic Receptors and Metabolism: Role in Development of Cardiovascular Disease. Front. Physiol..

[32] Depre C., Rider M.H., Veitch K., Hue L. (1993). Role of Fructose 2,6-Bisphosphate in the Control of Heart Glycolysis. J. Biol. Chem..

[33] Lawson J.W., Uyeda K. (1987). Effects of Insulin and Work on Fructose 2,6-Bisphosphate Content and Phosphofructokinase Activity in Perfused Rat Hearts. J. Biol. Chem..

[34] Harold K.M., Matsuzaki S., Pranay A., Zhu J., Faakye A., Humphries K.M. (2025). PFKFB2 Is Pivotal for Metabolic Flexibility and Differential Glucose Utilization. J. Am. Heart Assoc..

[35] Wang J., Xu J., Wang Q., Brainard R.E., Watson L.J., Jones S.P., Epstein P.N. (2013). Reduced Cardiac Fructose 2,6 Bisphosphate Increases Hypertrophy and Decreases Glycolysis Following Aortic Constriction. PLoS ONE.

[36] Packer M. (2023). Foetal Recapitulation of Nutrient Surplus Signalling by O-GlcNAcylation and the Failing Heart. Eur. J. Heart Fail..

[37] Laczy B., Fülöp N., Onay-Besikci A., Des Rosiers C., Chatham J.C. (2011). Acute Regulation of Cardiac Metabolism by the Hexosamine Biosynthesis Pathway and Protein O-GlcNAcylation. PLoS ONE.

[38] Tran D.H., May H.I., Li Q., Luo X., Huang J., Zhang G., Niewold E., Wang X., Gillette T.G., Deng Y. (2020). Chronic Activation of Hexosamine Biosynthesis in the Heart Triggers Pathological Cardiac Remodeling. Nat. Commun..

[39] Li Y., Xiong Z., Yan W., Gao E., Cheng H., Wu G., Liu Y., Zhang L., Li C., Wang S. (2020). Branched Chain Amino Acids Exacerbate Myocardial Ischemia/Reperfusion Vulnerability via Enhancing GCN2/ATF6/PPAR-α Pathway-Dependent Fatty Acid Oxidation. Theranostics.

[40] Wang W., Zhang F., Xia Y., Zhao S., Yan W., Wang H., Lee Y., Li C., Zhang L., Lian K. (2016). Defective Branched Chain Amino Acid Catabolism Contributes to Cardiac Dysfunction and Remodeling Following Myocardial Infarction. Am. J. Physiol. Heart Circ. Physiol..

[41] Schlueter N., de Sterke A., Willmes D.M., Spranger J., Jordan J., Birkenfeld A.L. (2014). Metabolic Actions of Natriuretic Peptides and Therapeutic Potential in the Metabolic Syndrome. Pharmacol. Ther..

[42] Gruden G., Landi A., Bruno G. (2014). Natriuretic Peptides, Heart, and Adipose Tissue: New Findings and Future Developments for Diabetes Research. Diabetes Care.

[43] Gupta D.K., Wang T.J. (2015). Natriuretic Peptides and Cardiometabolic Health. Circ. J. Off. J. Jpn. Circ. Soc..

[44] Reinke H., Asher G. (2019). Crosstalk between Metabolism and Circadian Clocks. Nat. Rev. Mol. Cell Biol..

[45] Glatz J.F.C., Heather L.C., Luiken J.J.F.P. (2024). CD36 as a Gatekeeper of Myocardial Lipid Metabolism and Therapeutic Target for Metabolic Disease. Physiol. Rev..

[46] Choi R.H., Tatum S.M., Symons J.D., Summers S.A., Holland W.L. (2021). Ceramides and Other Sphingolipids as Drivers of Cardiovascular Disease. Nat. Rev. Cardiol..

[47] Law B.A., Liao X., Moore K.S., Southard A., Roddy P., Ji R., Szulc Z., Bielawska A., Schulze P.C., Cowart L.A. (2018). Lipotoxic Very-Long-Chain Ceramides Cause Mitochondrial Dysfunction, Oxidative Stress, and Cell Death in Cardiomyocytes. FASEB J..

[48] Niemann B., Rohrbach S., Miller M.R., Newby D.E., Fuster V., Kovacic J.C. (2017). Oxidative Stress and Cardiovascular Risk: Obesity, Diabetes, Smoking, and Pollution: Part 3 of a 3-Part Series. J. Am. Coll. Cardiol..

[49] Marwick T.H., Ritchie R., Shaw J.E., Kaye D. (2018). Implications of Underlying Mechanisms for the Recognition and Management of Diabetic Cardiomyopathy. J. Am. Coll. Cardiol..

[50] Packer M. (2020). Longevity Genes, Cardiac Ageing, and the Pathogenesis of Cardiomyopathy: Implications for Understanding the Effects of Current and Future Treatments for Heart Failure. Eur. Heart J..

[51] Iacobini C., Vitale M., Haxhi J., Pesce C., Pugliese G., Menini S. (2022). Mutual Regulation between Redox and Hypoxia-Inducible Factors in Cardiovascular and Renal Complications of Diabetes. Antioxidants.

[52] Warbrick I., Rabkin S.W. (2019). Hypoxia-Inducible Factor 1-Alpha (HIF-1α) as a Factor Mediating the Relationship between Obesity and Heart Failure with Preserved Ejection Fraction. Obes. Rev. Off. J. Int. Assoc. Study Obes..

[53] Sousa Fialho M.d.L., Purnama U., Dennis K.M.J.H., Montes Aparicio C.N., Castro-Guarda M., Massourides E., Tyler D.J., Carr C.A., Heather L.C. (2021). Activation of HIF1α Rescues the Hypoxic Response and Reverses Metabolic Dysfunction in the Diabetic Heart. Diabetes.

[54] Mericskay M., Zuurbier C.J., Heather L.C., Karlstaedt A., Inserte J., Bertrand L., Kararigas G., Ruiz-Meana M., Maack C., Schiattarella G.G. (2025). Cardiac Intermediary Metabolism in Heart Failure: Substrate Use, Signalling Roles and Therapeutic Targets. Nat. Rev. Cardiol..

[55] Ritterhoff J., Tian R. (2023). Metabolic Mechanisms in Physiological and Pathological Cardiac Hypertrophy: New Paradigms and Challenges. Nat. Rev. Cardiol..

[56] Flam E., Arany Z. (2023). Metabolite Signaling in the Heart. Nat. Cardiovasc. Res..

[57] Acetylation in Cardiovascular Diseases: Molecular Mechanisms and Clinical Implications-PubMed. https://pubmed.ncbi.nlm.nih.gov/32413386/.

[58] Kane A.E., Sinclair D.A. (2018). Sirtuins and NAD+ in the Development and Treatment of Metabolic and Cardiovascular Diseases. Circ. Res..

[59] Carpenter B.J., Dierickx P. (2022). Circadian Cardiac NAD+ Metabolism, from Transcriptional Regulation to Healthy Aging. Am. J. Physiol. Cell Physiol..

[60] Preservation of Acyl Coenzyme a Attenuates Pathological and Metabolic Cardiac Remodeling Through Selective Lipid Trafficking-PubMed. https://pubmed.ncbi.nlm.nih.gov/30909726/.

[61] Telesca M., Masciovecchio V., Costantino S. (2026). Metabolic Rewiring through Succinate-GPR91 Signaling: A Fresh Perspective on HFpEF Energetics. Cardiovasc. Diabetol..

[62] Succinate-GPR91 Signaling Promotes Cardiomyocyte Metabolic Reprogramming and NAD+ Production to Alleviate HFpEF-PubMed. https://pubmed.ncbi.nlm.nih.gov/41353145/.

[63] Salt I.P., Hardie D.G. (2017). AMP-Activated Protein Kinase: An Ubiquitous Signaling Pathway with Key Roles in the Cardiovascular System. Circ. Res..

[64] Sharma A., Anand S.K., Singh N., Dwivedi U.N., Kakkar P. (2023). AMP-Activated Protein Kinase: An Energy Sensor and Survival Mechanism in the Reinstatement of Metabolic Homeostasis. Exp. Cell Res..

[65] Goul C., Peruzzo R., Zoncu R. (2023). The Molecular Basis of Nutrient Sensing and Signalling by mTORC1 in Metabolism Regulation and Disease. Nat. Rev. Mol. Cell Biol..

[66] Sciarretta S., Forte M., Frati G., Sadoshima J. (2018). New Insights Into the Role of mTOR Signaling in the Cardiovascular System. Circ. Res..

[67] Packer M. (2025). Intensity, Duration, and Context Dependency of the Responses to Nutrient Surplus and Deprivation Signaling in the Heart: Insights into the Complexities of Cardioprotection. Circulation.

[68] Zhu J., Marciniak S.J. (2026). GCN2 in Proteostasis: Structural Logic, Signalling Networks and Disease. FEBS J..

[69] Lu Z., Xu X., Fassett J., Kwak D., Liu X., Hu X., Wang H., Guo H., Xu D., Yan S. (2014). Loss of the Eukaryotic Initiation Factor 2α Kinase General Control Nonderepressible 2 Protects Mice from Pressure Overload-Induced Congestive Heart Failure without Affecting Ventricular Hypertrophy. Hypertension.

[70] Feng W., Lei T., Wang Y., Feng R., Yuan J., Shen X., Wu Y., Gao J., Ding W., Lu Z. (2019). GCN2 Deficiency Ameliorates Cardiac Dysfunction in Diabetic Mice by Reducing Lipotoxicity and Oxidative Stress. Free Radic. Biol. Med..

[71] Veera S., Tang F., Mourad Y., Kim S., Liu T., Li H., Wang Y., Warren J.S., Park J., Van C. (2024). A Transcriptional Regulatory Mechanism of Genes in the Tricarboxylic Acid Cycle in the Heart. J. Biol. Chem..

[72] Ramjiawan A., Bagchi R.A., Albak L., Czubryt M.P. (2013). Mechanism of Cardiomyocyte PGC-1α Gene Regulation by ERRα. Biochem. Cell Biol..

[73] Sakamoto T., Batmanov K., Wan S., Guo Y., Lai L., Vega R.B., Kelly D.P. (2022). The Nuclear Receptor ERR Cooperates with the Cardiogenic Factor GATA4 to Orchestrate Cardiomyocyte Maturation. Nat. Commun..

[74] Yamamoto T., Maurya S.K., Pruzinsky E., Batmanov K., Xiao Y., Sulon S.M., Sakamoto T., Wang Y., Lai L., McDaid K.S. (2023). RIP140 Deficiency Enhances Cardiac Fuel Metabolism and Protects Mice from Heart Failure. J. Clin. Investig..

[75] Jiang Y., Jiang J., Feng S., Zhu R. (2025). Advances in BRD Proteins in Cardiovascular Pathophysiology and Therapy: A Review. Int. J. Biol. Macromol..

[76] Martin R.D., Sun Y., MacKinnon S., Cuccia L., Pagé V., Hébert T.E., Tanny J.C. (2020). Differential Activation of P-TEFb Complexes in the Development of Cardiomyocyte Hypertrophy Following Activation of Distinct G Protein-Coupled Receptors. Mol. Cell. Biol..

[77] Mu J., Zhang D., Tian Y., Xie Z., Zou M.-H. (2020). BRD4 Inhibition by JQ1 Prevents High-Fat Diet-Induced Diabetic Cardiomyopathy by Activating PINK1/Parkin-Mediated Mitophagy in Vivo. J. Mol. Cell. Cardiol..

[78] Jia Y., Chang H.-C., Schipma M.J., Liu J., Shete V., Liu N., Sato T., Thorp E.B., Barger P.M., Zhu Y.-J. (2016). Cardiomyocyte-Specific Ablation of Med1 Subunit of the Mediator Complex Causes Lethal Dilated Cardiomyopathy in Mice. PLoS ONE.

[79] Cardiac Med1 Deletion Promotes Early Lethality, Cardiac Remodeling, and Transcriptional Reprogramming-PubMed. https://pubmed.ncbi.nlm.nih.gov/28159809/.

[80] Grueter C.E., van Rooij E., Johnson B.A., DeLeon S.M., Sutherland L.B., Qi X., Gautron L., Elmquist J.K., Bassel-Duby R., Olson E.N. (2012). A Cardiac microRNA Governs Systemic Energy Homeostasis by Regulation of MED13. Cell.

[81] Pepin M.E., Gong X., Schulze A., Backs J. (2026). Metabo-Epigenetic Circuits of Heart Failure: Chromatin-Modifying Enzymes as Determinants of Metabolic Plasticity. EMBO Mol. Med..

[82] Flowers B., Duric B. (2025). Metabolic-Epigenetic Interactions in Heart Failure: Current Understanding and Future Directions. Biochem. Biophys. Res. Commun..

[83] Laurette P., Gilsbach R. (2026). Cardiac Epigenome in Heart Development and Disease. Nat. Rev. Cardiol..

[84] Jia G., Hill M.A., Sowers J.R. (2018). Diabetic Cardiomyopathy: An Update of Mechanisms Contributing to This Clinical Entity. Circ. Res..

[85] Yun H.R., Singh M.K., Han S., Ranbhise J.S., Ha J., Kim S.S., Kang I. (2026). Biomarkers of Cardiac Metabolic Flexibility in Health, HFrEF and HFpEF. Int. J. Mol. Sci..

[86] Tran D.H., Wang Z.V. (2019). Glucose Metabolism in Cardiac Hypertrophy and Heart Failure. J. Am. Heart Assoc..

[87] Opie L.H., Knuuti J. (2009). The Adrenergic-Fatty Acid Load in Heart Failure. J. Am. Coll. Cardiol..

[88] Hue L., Taegtmeyer H. (2009). The Randle Cycle Revisited: A New Head for an Old Hat. Am. J. Physiol. Endocrinol. Metab..

[89] Zhelev Z., Aoki I., Lazarova D., Vlaykova T., Higashi T., Bakalova R. (2022). A “Weird” Mitochondrial Fatty Acid Oxidation as a Metabolic “Secret” of Cancer. Oxid. Med. Cell. Longev..

[90] Smith R.L., Soeters M.R., Wüst R.C.I., Houtkooper R.H. (2018). Metabolic Flexibility as an Adaptation to Energy Resources and Requirements in Health and Disease. Endocr. Rev..

[91] Tirichen H., Yaigoub H., Xu W., Wu C., Li R., Li Y. (2021). Mitochondrial Reactive Oxygen Species and Their Contribution in Chronic Kidney Disease Progression Through Oxidative Stress. Front. Physiol..

[92] Inferrera F., Marino Y., Genovese T., Cuzzocrea S., Fusco R., Di Paola R. (2025). Mitochondrial Quality Control: Biochemical Mechanism of Cardiovascular Disease. Biochim. Biophys. Acta Mol. Cell Res..

[93] Ježek P., Holendová B., Plecitá-Hlavatá L. (2020). Redox Signaling from Mitochondria: Signal Propagation and Its Targets. Biomolecules.

[94] Chen S., Li Q., Shi H., Li F., Duan Y., Guo Q. (2024). New Insights into the Role of Mitochondrial Dynamics in Oxidative Stress-Induced Diseases. Biomed. Pharmacother..

[95] Yuan T., Yang T., Chen H., Fu D., Hu Y., Wang J., Yuan Q., Yu H., Xu W., Xie X. (2019). New Insights into Oxidative Stress and Inflammation during Diabetes Mellitus-Accelerated Atherosclerosis. Redox Biol..

[96] Molecular and Cellular Mechanisms of Cardiovascular Disorders in Diabetes-PubMed. https://pubmed.ncbi.nlm.nih.gov/27230643/.

[97] Mercola J. (2025). Reductive Stress and Mitochondrial Dysfunction: The Hidden Link in Chronic Disease. Free Radic. Biol. Med..

[98] Salyer L.G., Wang Y., Ma X., Foryst-Ludwig A., Kintscher U., Chennappan S., Kontaridis M.I., McKinsey T.A. (2025). Modulating the Secretome of Fat to Treat Heart Failure. Circ. Res..

[99] Wallhaus T.R., Taylor M., DeGrado T.R., Russell D.C., Stanko P., Nickles R.J., Stone C.K. (2001). Myocardial Free Fatty Acid and Glucose Use after Carvedilol Treatment in Patients with Congestive Heart Failure. Circulation.

[100] Morciano G., Vitto V.A.M., Bouhamida E., Giorgi C., Pinton P. (2021). Mitochondrial Bioenergetics and Dynamism in the Failing Heart. Life.

[101] Werbner B., Tavakoli-Rouzbehani O.M., Fatahian A.N., Boudina S. (2023). The Dynamic Interplay between Cardiac Mitochondrial Health and Myocardial Structural Remodeling in Metabolic Heart Disease, Aging, and Heart Failure. J. Cardiovasc. Aging.

[102] Broeglin A., Riou A., Papassotiropoulos A., Eckert A., Grimm A. (2025). Mitochondrial Transplantation Increases Bioenergetics and Neurite Outgrowth in Healthy and P301Ltau-Expressing SH-SY5Y Cells. Mol. Neurobiol..

[103] Schulze P.C. (2009). Myocardial Lipid Accumulation and Lipotoxicity in Heart Failure. J. Lipid Res..

[104] Tanasescu M.-D., Rosu A.-M., Minca A., Rosu A.-L., Grigorie M.-M., Timofte D., Ionescu D. (2026). Metabolic Dysfunction at the Core: Revisiting the Overlap of Cardiovascular, Renal, Hepatic, and Endocrine Disorders. Life.

[105] Huang Y., Zhou B. (2023). Mitochondrial Dysfunction in Cardiac Diseases and Therapeutic Strategies. Biomedicines.

[106] Wróbel-Nowicka K., Wojciechowska C., Jacheć W., Zalewska M., Romuk E. (2024). The Role of Oxidative Stress and Inflammatory Parameters in Heart Failure. Medicina.

[107] Santulli G., Sabatelli G., Wang B., Savino M., Bruno F.P., Jankauskas S.S., Massaro A., Peluso C., Vicario M., Savino L. (2025). Interplay between Frailty and Cardiometabolic Disorders: From Pathophysiology to Clinical Implications. Cardiovasc. Diabetol..

[108] Marin C., Ramirez R., Delgado-Lista J., Yubero-Serrano E.M., Perez-Martinez P., Carracedo J., Garcia-Rios A., Rodriguez F., Gutierrez-Mariscal F.M., Gomez P. (2011). Mediterranean Diet Reduces Endothelial Damage and Improves the Regenerative Capacity of Endothelium. Am. J. Clin. Nutr..

[109] Goyal P., Balkan L., Ringel J.B., Hummel S.L., Sterling M.R., Kim S., Arora P., Jackson E.A., Brown T.M., Shikany J.M. (2021). The Dietary Approaches to Stop Hypertension (DASH) Diet Pattern and Incident Heart Failure. J. Card. Fail..

[110] Hummel S.L., Karmally W., Gillespie B.W., Helmke S., Teruya S., Wells J., Trumble E., Jimenez O., Marolt C., Wessler J.D. (2018). Home-Delivered Meals Postdischarge from Heart Failure Hospitalization. Circ. Heart Fail..

[111] Kraus W.E., Bhapkar M., Huffman K.M., Pieper C.F., Krupa Das S., Redman L.M., Villareal D.T., Rochon J., Roberts S.B., Ravussin E. (2019). 2 Years of Calorie Restriction and Cardiometabolic Risk (CALERIE): Exploratory Outcomes of a Multicentre, Phase 2, Randomised Controlled Trial. Lancet Diabetes Endocrinol..

[112] Weiss E.P., Fontana L. (2011). Caloric Restriction: Powerful Protection for the Aging Heart and Vasculature. Am. J. Physiol. Heart Circ. Physiol..

[113] Chandler P.D., Balasubramanian R., Paynter N., Giulianini F., Fung T., Tinker L.F., Snetselaar L., Liu S., Eaton C., Tobias D.K. (2020). Metabolic Signatures Associated with Western and Prudent Dietary Patterns in Women. Am. J. Clin. Nutr..

[114] O’Shea K.M., Khairallah R.J., Sparagna G.C., Xu W., Hecker P.A., Robillard-Frayne I., Des Rosiers C., Kristian T., Murphy R.C., Fiskum G. (2009). Dietary Omega-3 Fatty Acids Alter Cardiac Mitochondrial Phospholipid Composition and Delay Ca^2+^-Induced Permeability Transition. J. Mol. Cell. Cardiol..

[115] Yurista S.R., Chong C.-R., Badimon J.J., Kelly D.P., de Boer R.A., Westenbrink B.D. (2021). Therapeutic Potential of Ketone Bodies for Patients with Cardiovascular Disease: JACC State-of-the-Art Review. J. Am. Coll. Cardiol..

[116] Foster M.W., Riley J.M., Kaki P.C., Al Soueidy A., Aligholiazadeh E., Rame J.E. (2024). Metabolic Adaptation in Heart Failure and the Role of Ketone Bodies as Biomarkers. Curr. Heart Fail. Rep..

[117] Challa A.A., Hill B.G., Nystoriak M.A., Gouwens K.R., Kalra D.K. (2025). Ketone Bodies in Cardiovascular Disease: The Vasculature as a Therapeutic Target. JACC Basic Transl. Sci..

[118] Saris C.G.J., Timmers S. (2022). Ketogenic Diets and Ketone Suplementation: A Strategy for Therapeutic Intervention. Front. Nutr..

[119] Moreno-Vedia J., Llop D., Rodríguez-Calvo R., Plana N., Amigó N., Rosales R., Esteban Y., Girona J., Masana L., Ibarretxe D. (2023). Serum Branch-Chained Amino Acids Are Increased in Type 2 Diabetes and Associated with Atherosclerotic Cardiovascular Disease. Cardiovasc. Diabetol..

[120] Zhang J., Liu Z., Ni Y., Yu Y., Guo F., Lu Y., Wang X., Hao H., Li S., Wei P. (2024). Branched-Chain Amino Acids Promote Occurrence and Development of Cardiovascular Disease Dependent on Triglyceride Metabolism via Activation of the mTOR/SREBP-1/Betatrophin Pathway. Mol. Cell. Endocrinol..

[121] Bomer N., Pavez-Giani M.G., Grote Beverborg N., Cleland J.G.F., van Veldhuisen D.J., van der Meer P. (2022). Micronutrient Deficiencies in Heart Failure: Mitochondrial Dysfunction as a Common Pathophysiological Mechanism?. J. Intern. Med..

[122] Serra M., Mollace R., Ritorto G., Ussia S., Altomare C., Tavernese A., Preianò M., Palma E., Muscoli C., Mollace V. (2025). A Systematic Review of Thiamine Supplementation in Improving Diabetes and Its Related Cardiovascular Dysfunction. Int. J. Mol. Sci..

[123] Alarcón-Vieco E., Martínez-García I., Sequí-Domínguez I., Rodríguez-Gutiérrez E., Moreno-Herráiz N., Pascual-Morena C. (2023). Effect of Coenzyme Q10 on Cardiac Function and Survival in Heart Failure: An Overview of Systematic Reviews and Meta-Analyses. Food Funct..

[124] Vijver M.A.T., Bomer N., Verdonk R.C., van der Meer P., van Veldhuisen D.J., Dams O.C. (2024). Micronutrient Deficiencies in Heart Failure and Relationship with Exocrine Pancreatic Insufficiency. Nutrients.

[125] Perrone P., D’Angelo S. (2025). Gut Microbiota Modulation Through Mediterranean Diet Foods: Implications for Human Health. Nutrients.

[126] Nourazarain A., Vaziri Y. (2025). Nutrigenomics Meets Multi-Omics: Integrating Genetic, Metabolic, and Microbiome Data for Personalized Nutrition Strategies. Genes Nutr..

[127] Fayyaz K., Din M.S.U., Bashir H., Ahmad F., Barrow C.J., Khalid N. (2025). Personalized Nutrition Biomarkers and Dietary Strategies for Atherosclerosis Risk Management: A Systematic Review. Nutrients.

[128] Estruch R., Ros E., Salas-Salvadó J., Covas M.-I., Corella D., Arós F., Gómez-Gracia E., Ruiz-Gutiérrez V., Fiol M., Lapetra J. (2018). Primary Prevention of Cardiovascular Disease with a Mediterranean Diet Supplemented with Extra-Virgin Olive Oil or Nuts. N. Engl. J. Med..

[129] Delgado-Lista J., Alcala-Diaz J.F., Torres-Peña J.D., Quintana-Navarro G.M., Fuentes F., Garcia-Rios A., Ortiz-Morales A.M., Gonzalez-Requero A.I., Perez-Caballero A.I., Yubero-Serrano E.M. (2022). Long-Term Secondary Prevention of Cardiovascular Disease with a Mediterranean Diet and a Low-Fat Diet (CORDIOPREV): A Randomised Controlled Trial. Lancet.

[130] de Lorgeril M., Salen P., Martin J.L., Monjaud I., Delaye J., Mamelle N. (1999). Mediterranean Diet, Traditional Risk Factors, and the Rate of Cardiovascular Complications after Myocardial Infarction: Final Report of the Lyon Diet Heart Study. Circulation.

[131] Levitan E.B., Lewis C.E., Tinker L.F., Eaton C.B., Ahmed A., Manson J.E., Snetselaar L.G., Martin L.W., Trevisan M., Howard B.V. (2013). Mediterranean and DASH Diet Scores and Mortality in Women with Heart Failure: The Women’s Health Initiative. Circ. Heart Fail..

[132] Arayici M.E., Kilic M.E., Yilmaz M.B. (2025). High and Low Adherence to Mediterranean and DASH Diet Patterns and the Risk of Heart Failure: A Meta-Analysis of Observational Studies. Life.

[133] Kirkham A.A., Beka V., Prado C.M. (2021). The Effect of Caloric Restriction on Blood Pressure and Cardiovascular Function: A Systematic Review and Meta-Analysis of Randomized Controlled Trials. Clin. Nutr..

[134] Abdellatif M., Sedej S., Carmona-Gutierrez D., Madeo F., Kroemer G. (2018). Autophagy in Cardiovascular Aging. Circ. Res..

[135] Kodur N., Yurista S., Province V., Rueth E., Nguyen C., Tang W.H.W. (2023). Ketogenic Diet in Heart Failure: Fact or Fiction?. JACC Heart Fail..

[136] Belardo D., Michos E.D., Blankstein R., Blumenthal R.S., Ferdinand K.C., Hall K., Klatt K., Natajaran P., Ostfeld R.J., Reddy K. (2022). Practical, Evidence-Based Approaches to Nutritional Modifications to Reduce Atherosclerotic Cardiovascular Disease: An American Society for Preventive Cardiology Clinical Practice Statement. Am. J. Prev. Cardiol..

[137] Mirmiran P., Bahadoran Z., Gaeini Z. (2021). Common Limitations and Challenges of Dietary Clinical Trials for Translation into Clinical Practices. Int. J. Endocrinol. Metab..

[138] Shakoor A., van Maarschalkerwaart W.A., Schaap J., de Boer R.A., van Mieghem N.M., Boersma E.H., van Heerebeek L., Brugts J.J., van der Boon R.M.A. (2025). Socio-Economic Inequalities and Heart Failure Morbidity and Mortality: A Systematic Review and Data Synthesis. ESC Heart Fail..

[139] Yin Y., Li H., Hu Q., Wu D. (2026). BCAA Metabolic Dyshomeostasis in Cardiovascular Disease: Pathogenic Mechanisms and Intervention. Am. J. Cardiovasc. Drugs Drugs Devices Interv..

[140] Adafer R., Messaadi W., Meddahi M., Patey A., Haderbache A., Bayen S., Messaadi N. (2020). Food Timing, Circadian Rhythm and Chrononutrition: A Systematic Review of Time-Restricted Eating’s Effects on Human Health. Nutrients.

[141] Ho K.L., Karwi Q.G., Wagg C., Zhang L., Vo K., Altamimi T., Uddin G.M., Ussher J.R., Lopaschuk G.D. (2021). Ketones Can Become the Major Fuel Source for the Heart but Do Not Increase Cardiac Efficiency. Cardiovasc. Res..

[142] Sun W., Lin R., Li Y., Yao Y., Lu B., Yu Y. (2025). Circulating Branched-Chain Amino Acids and the Risk of Major Adverse Cardiovascular Events in the UK Biobank. Front. Endocrinol..

[143] Yu D., Richardson N.E., Green C.L., Spicer A.B., Murphy M.E., Flores V., Jang C., Kasza I., Nikodemova M., Wakai M.H. (2021). The Adverse Metabolic Effects of Branched-Chain Amino Acids Are Mediated by Isoleucine and Valine. Cell Metab..

[144] Li Z., Xia H., Sharp T.E., LaPenna K.B., Elrod J.W., Casin K.M., Liu K., Calvert J.W., Chau V.Q., Salloum F.N. (2022). Mitochondrial H2S Regulates BCAA Catabolism in Heart Failure. Circ. Res..

[145] O’Connell T.D., Block R.C., Huang S.P., Shearer G.C. (2017). Ω3-Polyunsaturated Fatty Acids for Heart Failure: Effects of Dose on Efficacy and Novel Signaling through Free Fatty Acid Receptor 4. J. Mol. Cell. Cardiol..

[146] Gorica E., Geiger M.A., Di Venanzio L., Atzemian N., Kleeberger J.A., Grigorian D., Mongelli A., Emini Veseli B., Mohammed S.A., Ruschitzka F. (2025). Cardiometabolic Heart Failure with Preserved Ejection Fraction: From Molecular Signatures to Personalized Treatment. Cardiovasc. Diabetol..

[147] Kilfoil P.J., Lotteau S., Zhang R., Yue X., Aynaszyan S., Solymani R.E., Cingolani E., Marbán E., Goldhaber J.I. (2020). Distinct Features of Calcium Handling and β-Adrenergic Sensitivity in Heart Failure with Preserved versus Reduced Ejection Fraction. J. Physiol..

[148] Knutson K.L., Dixon D.D., Grandner M.A., Jackson C.L., Kline C.E., Maher L., Makarem N., Martino T.A., St-Onge M.-P., Johnson D.A. (2025). Role of Circadian Health in Cardiometabolic Health and Disease Risk: A Scientific Statement from the American Heart Association. Circulation.

[149] Palomar-Cros A., Andreeva V.A., Fezeu L.K., Julia C., Bellicha A., Kesse-Guyot E., Hercberg S., Romaguera D., Kogevinas M., Touvier M. (2023). Dietary Circadian Rhythms and Cardiovascular Disease Risk in the Prospective NutriNet-Santé Cohort. Nat. Commun..

[150] Chulenbayeva L., Issilbayeva A., Sailybayeva A., Bekbossynova M., Kozhakhmetov S., Kushugulova A. (2025). Short-Chain Fatty Acids and Their Metabolic Interactions in Heart Failure. Biomedicines.

[151] Du W., Zou Z.-P., Ye B.-C., Zhou Y. (2025). Gut Microbiota and Associated Metabolites: Key Players in High-Fat Diet-Induced Chronic Diseases. Gut Microbes.

[152] Zhu R., Dong Y., Guo J., He J., Chen H., Wang R., Ren F., Raben A., Martinez J.A. (2025). From Prediction of Personalized Metabolic Responses to Foods to Computational Nutrition: Concepts of an Emerging Interdisciplinary Field. Clin. Nutr..

[153] Trouwborst I., Gijbels A., Jardon K.M., Siebelink E., Hul G.B., Wanders L., Erdos B., Péter S., Singh-Povel C.M., de Vogel-van den Bosch J. (2023). Cardiometabolic Health Improvements upon Dietary Intervention Are Driven by Tissue-Specific Insulin Resistance Phenotype: A Precision Nutrition Trial. Cell Metab..

